# Integrated Multi-Omics Analysis Reveals an HCMV-Associated Late-Gene Signature Associated with Poor Survival in Pediatric Group 3 Medulloblastoma

**DOI:** 10.3390/biomedicines14061328

**Published:** 2026-06-11

**Authors:** Maria F. Stierle, Martin U. Schuhmann, Jens Schittenhelm, Martin Ebinger

**Affiliations:** 1 Section of Pediatric Neurosurgery, Department of Neurosurgery and Neurotechnology, University Hospital Tübingen, 72076 Tübingen, Germany; 2 Department of Neuropathology, Institute of Pathology and Neuropathology, University Hospital Tübingen, 72076 Tübingen, Germany; 3 Department of Hematology and Oncology, University Children’s Hospital, University Hospital Tübingen, 72076 Tübingen, Germany

**Keywords:** human cytomegalovirus, HCMV-associated signal, medulloblastoma, group 3, multi-omics, survival, pediatric brain tumors, oncomodulation, viral-associated molecular signatures

## Abstract

**Background:** Previous work from our group demonstrated an association between immunohistochemical detection of Human cytomegalovirus (HCMV) late antigen and poor event-free survival (EFS) in pediatric medulloblastoma. Whole-genome sequencing (WGS) further identified increased abundance of HCMV-aligned reads at the *UL88* locus, particularly in Group 3 tumors, a molecular subgroup associated with aggressive clinical behavior and poor prognosis. **Methods:** We performed an integrated multi-omics analysis of pediatric medulloblastoma using WGS (*n* = 39) and RNA sequencing (RNA-seq; *n* = 28) datasets. RNA-seq data were filtered using stringent alignment criteria (MAPQ ≥ 20) and compared with fetal brain *(n* = 12), adult brain (*n* = 12), and HCMV-infected cell culture controls (*n* = 3). Only high-confidence uniquely aligned reads were retained to reduce nonspecific and multi-mapped viral alignments. Sequencing reads were aligned to the HCMV Merlin reference genome (NC_006273.2) using a standardized analytical pipeline. A subset of 28 cases with matched tumor WGS, tumor RNA-seq, and germline WGS data was used for integrated multi-omics analyses. Orthogonal validation analyses were performed in Group 3 tumors using independent genomic and transcriptomic approaches. Exploratory survival analyses were conducted in a combined cohort (*n* = 84) integrating genomic and immunohistochemical datasets. **Results:** Recurrent low-level HCMV-aligned molecular signals were identified across medulloblastoma datasets. Reads aligning to *UL76*, *UL88*, and *UL99* were the most consistently detected HCMV-associated late-gene signals across RNA-seq and WGS datasets. A composite HCMV late-gene signature (*UL76–UL88–UL99*) showed higher levels in Group 3 tumors than in other molecular subgroups (*p* < 0.05 in WGS analyses). Orthogonal analyses demonstrated concordant low-level HCMV-associated genomic and transcriptomic signals enriched in tumors with *MYC*-associated activation and chromosome 17 imbalance. In the combined cohort (*n* = 84), elevated HCMV-associated signal assessed by immunohistochemistry and genomic profiling was associated with reduced EFS (median 55 vs. 147 months; log-rank *p* < 0.001). The subgroup classified as HCMV-high Group 3 demonstrated the strongest association with adverse outcome in exploratory multivariable analyses (HR = 6.43, *p* = 0.002). **Conclusions:** This study identifies recurrent low-level HCMV-associated genomic and transcriptomic signals across pediatric medulloblastoma datasets, with preferential enrichment in biologically aggressive Group 3 tumors. Although the extremely low abundance of viral-aligned reads precludes definitive evidence of productive viral infection, the reproducible detection of HCMV-associated molecular signatures across independent sequencing platforms supports further investigation into a potential oncomodulatory association in pediatric medulloblastoma. Additional validation using optimized viral detection methodologies, independent cohorts, and mechanistic studies will be necessary to clarify the biological and clinical significance of these findings.

## 1. Introduction

In a recent study, we identified a correlation between immunohistochemical detection of the HCMV late antigen and poorer event-free survival in a cohort of 45 pediatric patients with medulloblastoma from Tübingen, Germany [1]. In addition, WGS analysis revealed an increased abundance of HCMV-aligned reads at the *UL88* locus, which encodes a late tegument protein, particularly in Group 3 medulloblastomas, a molecular subgroup associated with poor prognosis [1,2].

HCMV genes *UL76*, *UL88*, and *UL99* have been associated with immune evasion, inflammation, and enhanced cell survival, which may influence tumor biology. These genes are classified as late genes, with peak expression occurring after viral DNA replication. Beyond their role in viral assembly, they contribute to immune evasion, inflammation, and enhanced cell survival [3,4,5,6].

*UL76* encodes the pUL76 protein, which has been reported to induce IL-8 expression by activating the NF-κB pathway through the DNA damage response. IL-8 is a key mediator of angiogenesis, tumor cell migration, and survival. In addition to its role in late-stage infection and its association with the viral envelope and tegument, UL76 has been linked to chromosomal aberrations, DNA damage, genomic instability, and G2/M cell cycle arrest in infected cells [3,7].

*UL88* encodes pUL88, a tegument protein that antagonizes innate immune signaling. Specifically, it has been reported to promote degradation of myeloid differentiation primary response protein 88 (MyD88), a key adapter molecule in Toll-like receptor signaling pathways, thereby potentially impairing immune detection and facilitating immune evasion. This mechanism may support viral persistence and promote tumor cell survival within an inflammatory microenvironment. *UL88* is expressed during late stages of infection and is also implicated in viral dissemination [6].

*UL99* encodes pp28, a structural protein essential for cytoplasmic viral assembly. Although its direct oncogenic role is less well-defined than that of *UL76* and *UL88*, *UL99* has been associated with efficient viral replication and persistence, which, in turn, may promote chronic inflammation and oncomodulatory effects within the tumor microenvironment [3,4,6].

Together, *UL76*, *UL88*, and *UL99* were selected as a putative HCMV-associated late-gene signature based on recurrent detection across datasets. Beyond their roles in viral replication, these genes may be associated with a tumor-supportive environment characterized by immunosuppression, chronic inflammation, and enhanced cellular adaptability [3,4,5,6,8]. In this study, we performed an integrated multi-omics analysis to investigate whether HCMV-aligned molecular signals correlate with molecular subgroups and clinical outcome in pediatric medulloblastoma.

## 2. Materials and Methods

### 2.1. Data Sources

Tumor sequencing data from the German pediatric medulloblastoma cohort were obtained from the European Genome-Phenome Archive (EGA; EGAD00001000122), including WGS data for 39 cases. Molecular subgroup classification, clinical, and survival data were retrieved from previously published cohorts [2,9].

RNA-seq data from an independent pediatric medulloblastoma cohort (*n* = 28, EGAD00001000328) were used for transcriptomic and integrative multi-omics analyses.

Control datasets included single-nucleus RNA-seq (snRNA-seq) data from adult human brain tissue (*n* = 12; ENA: PRJEB72935), RNA-seq data from human fetal brain (*n* = 12; EGAD00001004363), and RNA-seq data from HCMV-infected cell cultures (HCMV TB40E, *n* = 3; GEO; GSE73853), including neuronal progenitor cells (NPCs) and human foreskin fibroblasts (HFFs) [NPC-infected: GSM1904496, GSM1904495; HFF-infected: GSM1904490].

To establish a robust threshold for HCMV detection, non-tumor control samples (fetal and brain tissues) were used to set baseline viral alignment levels. These control datasets were compared with infected cell cultures and tumor RNA-seq datasets. Two complementary statistical approaches were used to define positivity thresholds: mean + 2 standard deviations and the 95th percentile of the control distributions, yielding concordant cut-offs. RNA-seq data were filtered to retain only high-confidence alignments.

### 2.2. Study Design and Multi-Omics Integration

A stepwise analytical framework was applied. Transcriptomic analyses were initially performed using RNA-seq data (*n* = 28), followed by genomic analyses using WGS data (*n* = 39). Subsequently, integrated multi-omics analyses were conducted using matched genomic and transcriptomic datasets.

For integrative multi-omics analyses, a subset of 28 cases with matched tumor WGS, germline (blood) WGS, and RNA-seq data was included. HCMV gene status was determined by comparing the mean log_2_(TPM + 1) values from RNA-seq and the mean log_2_(NVA + 1) values from tumor WGS against global 75th-percentile (p75) thresholds. Based on combined transcriptomic and genomic evidence, genes were classified as RNA+/DNA+ when both signals exceeded the threshold, RNA−/DNA+ when only the genomic signal exceeded the threshold, RNA+/DNA− when only the transcriptomic signal exceeded the threshold, and RNA−/DNA− when neither signal exceeded the threshold.

### 2.3. Viral Sequence Alignment and Quantification

Sequence reads were aligned to the human cytomegalovirus reference genome (HCMV strain Merlin; NC_006273.2) using Bowtie2 (version 2.5.4; Johns Hopkins University, Baltimore, MD, USA). The aligned reads were then processed with SAMtools (version 1.22.1; Wellcome Sanger Institute, Cambridge, UK) for sorting, indexing, and downstream analysis.

Gene-level viral transcript abundance for RNA-seq was quantified from aligned reads and normalized as transcripts per million (TPM). Expression values were transformed using log_2_(TPM + 1) to reduce skewness and stabilize variance. For the WGS-derived analysis, locus-level viral abundance was quantified as normalized viral abundance (NVA), calculated as gen length-normalized per million mapped viral reads, based on viral read counts mapped to HCMV genes. NVA values were subsequently transformed using log_2_(NVA + 1). This normalization accounts for differences in gene length and sequencing depth, enabling robust comparisons of viral signal across samples. A standardized pipeline was implemented across all datasets to ensure consistent alignment, quantification, and normalization of viral transcripts.

### 2.4. HCMV Viral Signal and Late-Locus Signature

Samples were considered to show evidence of HCMV signal if at least one viral locus exceeded a threshold of TPM > 10 in RNA-seq or NVA > 10 in WGS data. This threshold was used exclusively to detect the viral signal, whereas median composite Z-scores were used for stratification analyses.

The HCMV late-locus signature was defined by mapping reads to *UL76*, *UL88*, and *UL99*. For RNA-seq data, gene-level abundance was quantified as TPM and then transformed to log_2_(TPM + 1). For WGS data, viral locus abundance was quantified as NVA and then transformed to log_2_(NVA + 1).

For each locus, mean values were computed across all relevant transcript annotations within each sample. A composite HCMV late-locus signature score (*UL76–UL88–UL99*) was computed by averaging log_2_(TPM + 1) values from RNA-seq and log_2_(NVA + 1) values from WGS.

To enable cross-sample comparability, viral transcript expression for RNA-seq [log_2_ (TPM+1)] and viral locus signal for WGS [log_2_(NVA+1)] values were standardized to Z-scores across the cohort.

Samples were stratified into high- and low-signature groups based on the median composite Z-score. Values above the median were classified as “high,” and those below as “low signature” groups.

### 2.5. Statistical Analysis

All statistical analyses were performed using Python (version 3.10) and IBM SPSS Statistics (version 31.0). Differences in viral gene transcript expression in RNA-seq and in viral genomic signal in WGS between subgroups were assessed with the Kruskal–Wallis test for non-normally distributed data, followed by appropriate post hoc pairwise comparisons. For normally distributed variables, one-way ANOVA with post hoc tests was used. Survival analyses were conducted using Kaplan–Meier estimates and the log-rank test. Multivariate Cox proportional hazards models evaluated the prognostic impact of viral gene expression in RNA-seq and of viral locus signal in WGS across subgroups. Patients were stratified into HCMV-high Group 3 tumors (defined as Group 3 with an HCMV late-gene signature ≥ the median composite Z-score) for Cox analysis. Data visualization, including heatmaps, box plots, and forest plots, was performed using Python scientific libraries. A two-sided *p*-value of *p* < 0.05 was considered statistically significant.

### 2.6. Data Processing and Quality Control

Standard preprocessing steps were performed before downstream analyses. For RNA-seq datasets, raw paired-end FASTQ files were aligned directly to the HCMV reference genome (NC_006273.2) using Bowtie2 (version 2.5.4; Johns Hopkins University, Baltimore, MD, USA) with the --very-sensitive-local preset.

For WGS datasets, pre-aligned BAM files mapped to the human reference genome (GRCh37/hg19) were used as input. Reads were extracted and converted to FASTQ format prior to viral re-alignment, following a host-depletion strategy.

All alignments were processed using SAMtools (version 1.22.1; Wellcome Sanger Institute, Cambridge, UK) for sorting, indexing, and quality-control procedures. Initial detection of the HCMV signal was performed at the read level by calculating the overall viral alignment rate, with analyses restricted to high-confidence alignments (MAPQ ≥ 20).

For transcript-level analyses, reads were assigned to annotated HCMV transcripts to generate raw counts, which were then normalized to TPM and log_2_-transformed (TPM + 1). 

To define biologically relevant viral RNA-seq expression across tumors relative to controls, two complementary thresholds were applied: (i) a data-driven cutoff based on the distribution of control samples (mean + 2 standard deviations and the 95th percentile), and (ii) an expression-based cutoff (TPM > 10) to reduce low-level background transcription. (iii) Only high-confidence viral alignments (MAPQ > 20) were retained for downstream analysis. 

#### Orthogonal Validation Analysis in Group 3 Tumors

Raw RNA-seq reads (FASTQ) from Group 3 medulloblastoma samples were analyzed using a combined reference genome comprising the human reference genome (hg19) and the HCMV reference genome (NC_006273.2). Reads mapping to the HCMV reference genome were quantified across all samples. In parallel, genomic regions associated with Group 3 medulloblastoma biology were extracted from aligned RNA-seq datasets to evaluate tumor-associated genomic context.

For WGS analyses, previously aligned BAM files mapped to the human reference genome (hg19) were evaluated to assess genomic regions associated with Group 3 medulloblastoma biology. To further investigate the viral-associated signal, unmapped reads from the human genome alignments were extracted and re-aligned against the HCMV reference genome. High-confidence viral alignments were retained for downstream analyses and reads mapping to the HCMV reference genome were quantified across all samples.

### 2.7. Multi-Omics Integration and Latent Viral-Associated Signal Score

For integrative analyses, Z-scores for RNA-seq and WGS-derived HCMV-associated signals were computed across samples. A latent viral signal score was derived using principal component analysis (PCA), integrating standardized RNA and WGS features from tumor samples. The first principal component was used as a composite representation of the overall HCMV-associated molecular signal, capturing shared variance across genomic and transcriptomic HCMV-aligned features.

## 3. Results

### 3.1. Study Cohort

We analyzed data from pediatric medulloblastoma cases (≤19 years old). We first assessed HCMV-associated transcriptional signal in RNA-seq datasets (*n* = 28), then evaluated HCMV-associated genomic signal in WGS datasets (*n* = 39), and finally performed an integrated analysis using matched genomic and transcriptomic datasets (*n* = 28) [Tumor WGS, tumor RNA-seq, and germline (blood) WGS].

### 3.2. RNA-Seq Analysis

RNA-seq datasets from 28 medulloblastoma tumors were analyzed (mean age 7.57 years; SD = 3.9). 50% male (14/28). Molecular subgroups included WNT (3/28, 11%), SHH (7/28, 25%), Group 3 (9/28, 32%), and Group 4 (9/28, 32%).

Controls included fetal brain (*n* = 12), adult brain (*n* = 12), and HCMV-infected cell cultures (*n* = 3).

#### 3.2.1. HCMV-Aligned Transcriptomic Signal in Tumors and Control Samples

Low-level HCMV-aligned reads were detected across tumor samples (*n* = 28), with counts ranging from 505 to 4934 reads per sample (4.6 × 10^−6^ to 5.3 × 10^−5^ of total reads). These alignment rates were generally higher than those in control samples, including fetal and normal brain tissues (~10^−7^ to 10^−6^).

Most tumor samples showed HCMV-aligned reads above predefined background thresholds (mean + 2 SD and 95th percentile), whereas such signals were infrequent in control tissues. In contrast, HCMV-infected cell cultures exhibited consistently higher alignment rates (17–48%), supporting the alignment pipeline’s analytical performance (see Supplementary Table S1).

Although transcripts such as gp133 and gp165 were detected in the unfiltered data, they were absent after filtering for MAPQ ≥ 20, indicating low-confidence or multi-mapped reads. In contrast, *UL76*, *UL88*, and *UL99* remained among the most frequently detected aligned transcripts after filtering.

After screening (MAPQ ≥ 20), all tumor samples (*n* = 28) showed detectable HCMV-aligned reads with TPM > 10, with a mean of 14.0 ± 2.7 transcripts per sample (range 10–20). In total, twenty-eight HCMV-associated transcripts/loci were identified, with recurrent alignment to *UL76*, *UL88*, and *UL99* loci (TPM > 100) (see Supplementary Table S2).

#### 3.2.2. HCMV Late-Gene Signature in RNA-Seq

Kruskal–Wallis analysis demonstrated a significant difference in the HCMV late-gene signature (*UL76*, *UL88*, *UL99*) across tumor, control, and infected cell culture samples (*p* < 0.001).

**Figure 1:**
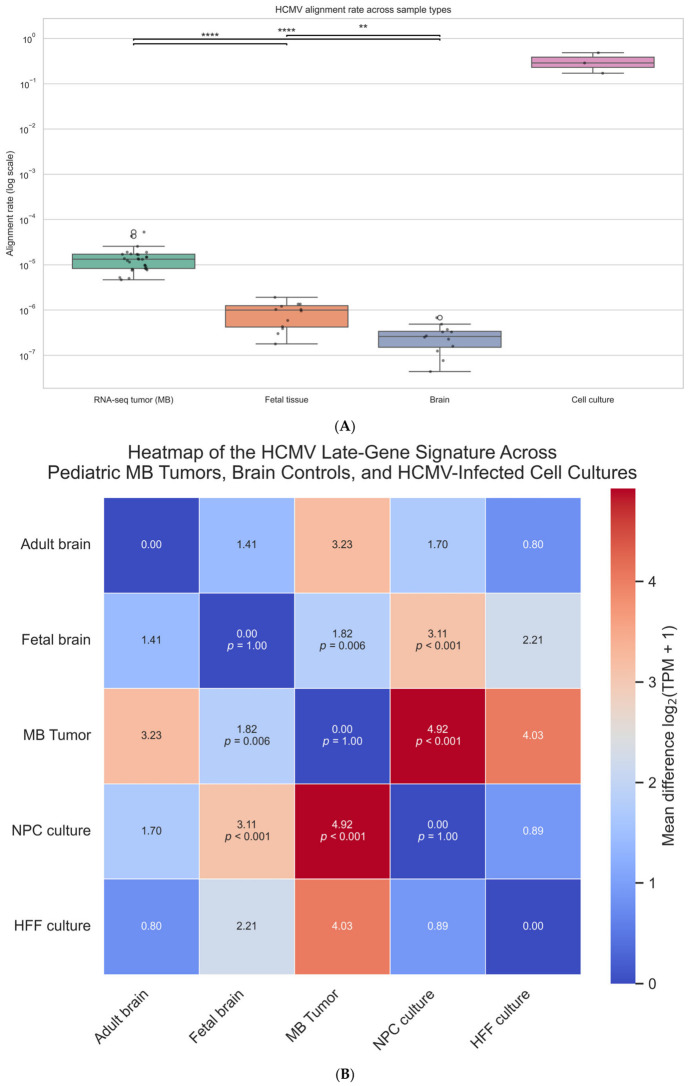
HCMV alignment rates and late-gene transcriptional signature across RNA-seq tumors compared with controls. (**A**) Kruskal–Wallis analysis revealed significant differences in HCMV alignment rates among sample groups (*p* = 1.31 × 10^−9^). Post hoc pairwise comparisons using Dunn’s test with Bonferroni correction showed clear stratification across conditions. Brain and fetal tissues did not differ significantly (*p* = 1.0), whereas both groups differed significantly from RNA-seq tumor (MB) samples (*p* < 0.01) and cell culture models (*p* < 0.001). Tumor (MB) samples also differed significantly from fetal and brain tissues but not from cell culture (*p* = 0.667), indicating partial overlap in viral signal distributions between tumor-derived RNA-seq data and in vitro infection systems. Overall, these results indicate a gradient of HCMV abundance, from background levels in neural tissues to elevated signals in tumor and cell culture conditions. Asterisks denote statistically significant differences (** *p* < 0.01, **** *p* < 0.001). (**B**) Heatmap of pairwise log_2_ fold changes of a composite HCMV late-gene expression signature (*UL76*, *UL88*, *UL99*), calculated as the mean expression across genes. Values represent relative differences between group-level means, with positive values indicating higher signature activity in the row group compared with the column group. Tumor samples exhibit a distinct HCMV-aligned transcriptional pattern compared with neural tissues and in vitro HCMV infection models. Quantitative analysis shows that tumor-derived viral transcript levels are significantly higher than in fetal brain tissue (log_2_FC = 1.82, *p* = 0.006) but lower than in in vitro infection systems, including human foreskin fibroblasts (HFF; log_2_FC = 4.03) and neural progenitor cells (NPC; log_2_FC = 4.92, *p* < 0.001). Adult brain samples show intermediate levels (log_2_FC = 3.23), suggesting a low-level HCMV-aligned transcriptional signal.

Tumor samples showed significantly higher composite expression compared to fetal brain (mean rank 38.46 vs. 16.42; adjusted *p* = 0.001) and adult brain (mean rank 38.46 vs. 8.67; adjusted *p* < 0.001). Although tumor samples showed increased HCMV-associated signal relative to neural controls, transcript levels remained substantially lower than those observed in infected cell culture systems, indicating a low-level HCMV-associated molecular signal rather than patterns consistent with productive viral infection (Figure 1).

Subgroup analyses showed that SHH, Group 3, and Group 4 tumors exhibited significantly higher HCMV-associated late-gene signal compared with normal brain controls (adjusted *p* < 0.05), whereas WNT tumors did not demonstrate significant differences (Supplementary Material S1).

Across medulloblastoma molecular subgroups, Group 3 tumors showed a trend toward higher composite expression of the HCMV-associated late-gene signature (*UL76*, *UL88*, *UL99*) than WNT tumors (mean rank: 17.23 vs. 7.33). However, these differences did not reach statistical significance following multiple-comparison correction (Kruskal–Wallis *p* = 0.175; adjusted pairwise *p* = 0.428) (Supplementary Material S2).

#### 3.2.3. Survival Analysis: RNA-Seq Tumor Cohort

Mean progression-free survival (PFS) was 64.2 months (95% CI: 54–73) and mean overall survival (OS) was 66.7 months (95% CI: 58–75). Recurrence and mortality rates were 18% (5/28) and 14% (4/28), respectively.

Kaplan–Meier analysis demonstrated that a high HCMV late-gene signature (composite ≥ median) was significantly associated with poorer PFS (*p* = 0.018) and OS (*p* = 0.043). Notably, all cases with recurrence (*n* = 5) and death (*n* = 4) exhibited a high viral signature. Furthermore, the HCMV-high Group 3 showed poorer EFS (43 vs. 60, *p* = 0.003) and OS (*p* < 0.001) than the other groups.

### 3.3. Whole Genome Sequence Analysis

**Figure 2:**
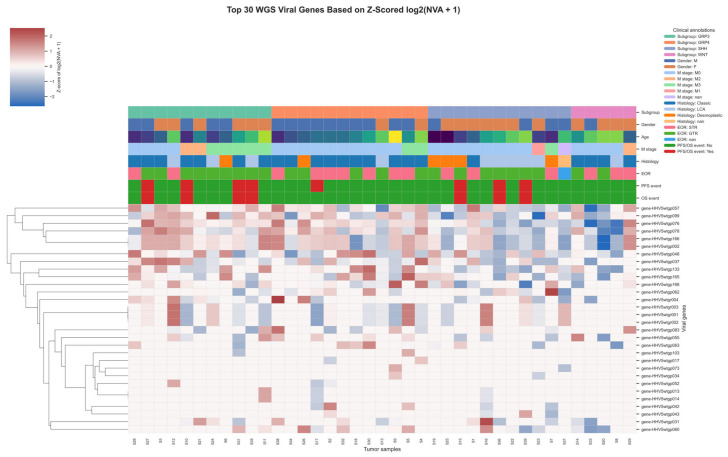
Genomic distribution of HCMV-associated signal across the WGS medulloblastoma cohort. Heatmap showing the top 30 most variable HCMV-associated loci based on z-score normalized log_2_-transformed normalized viral abundance values [log_2_(NVA + 1)] across 39 medulloblastoma whole-genome sequencing (WGS) samples. Hierarchical clustering was performed for both samples and viral loci to visualize relative patterns of HCMV-aligned genomic signal across the cohort. Samples are annotated according to molecular subgroup (Group 3, turquoise; Group 4, orange; SHH, violet; WNT, pink) and available clinical variables, including sex, age, metastatic stage (M0 vs. M1–M3), histological subtype, extent of resection (EOR), gross total resection (GTR), subtotal resection (STR), progression-free survival (PFS) event, and overall survival (OS) event. The heatmap demonstrates heterogeneous low-level HCMV-associated genomic signal across medulloblastoma samples, with recurrent enrichment of selected loci, including *UL76*, *UL88*, and *UL99*, particularly within subsets of Group 3 and Group 4 tumors. These findings support subgroup-associated variability in HCMV-aligned genomic signatures rather than evidence of uniform or high-level viral genomic abundance. Nan = not available data.

The WGS cohort comprised 39 pediatric medulloblastoma cases (mean age 8.33 years, range 1–17 years), 51% of which were male. Molecular subgroup distribution was WNT (5/39, 13%), SHH (11/39, 28%), Group 3 (11/39, 28%), and Group 4 (12/39, 31%). Histology was predominantly classic (22/39, 56%), followed by large cell/anaplastic (10/39, 26%) and desmoplastic (6/39, 15%). Metastatic disease was present in 31% of cases (12/39), and gross total resection was achieved in 60% (23/39). Recurrence and mortality rates were 21% (8/39) and 18% (7/39), respectively (Figure 2).

#### 3.3.1. HCMV Gene Viral Signal in WGS Tumors

Low-level HCMV-aligned genomic signal (NVA > 10) was detected across WGS samples, with a mean of 16.25 viral loci detected per sample (range 3–25). Across the cohort, fifty-one HCMV-associated loci had detectable aligned reads. Among these, *UL76*, *UL88*, and *UL99* were recurrently detected across multiple samples, consistent with observations from the RNA-seq analyses performed under stringent filtering conditions.

The relative abundance of HCMV-aligned reads varied across molecular subgroups. Overall subgroup differences approached statistical significance (Kruskal–Wallis *p* = 0.052). Group 4 tumors showed a higher HCMV-associated genomic signal than WNT tumors (adjusted *p* = 0.042), whereas Group 3 tumors showed a similar trend that did not remain significant after correction for multiple testing.

**Figure 3:**
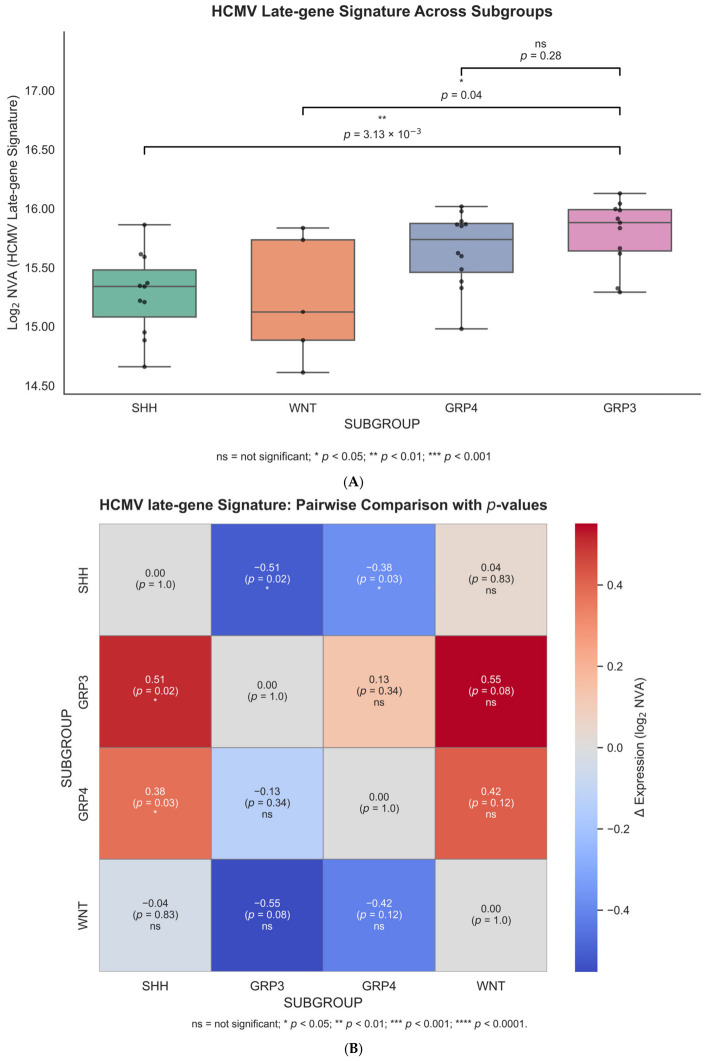
Subgroup-associated enrichment of the HCMV late-gene signature in the WGS cohort. Whole-genome sequencing (WGS) analysis of 39 pediatric medulloblastoma samples demonstrated subgroup-associated differences in the composite HCMV-associated late-gene signature derived from *UL76*, *UL88*, and *UL99* loci [mean log_2_(NVA + 1)]. (**A**) Upper panel: Boxplots comparing the distribution of the composite HCMV-associated late-gene signal across molecular subgroups. Kruskal–Wallis analysis showed significant overall differences among subgroups (*p* = 0.042). Group 3 tumors exhibited higher composite signal than SHH tumors (mean ranks: 27.45 vs. 12.00; unadjusted *p* = 0.004; adjusted *p* = 0.011). A similar trend was observed between Group 3 (GRP3) and WNT tumors, although this difference was not statistically significant after correction for multiple testing (unadjusted *p* = 0.014, adjusted *p* = 0.086). (**B**) Lower panel: Comparative heatmap of subgroup-level composite HCMV-associated late-gene signal demonstrating relative enrichment in Group 3 tumors compared with SHH and WNT tumors. Fold-change analyses showed increased signal in Group 3 relative to SHH (fold change = 0.51, *p* = 0.003) and WNT tumors (fold change = 0.55, *p* = 0.038). Overall, these findings indicate heterogeneous but recurrent low-level enrichment of HCMV-associated genomic signal across medulloblastoma subgroups, with the strongest relative enrichment observed in Group 3 tumors. GRP3 = Group 3 MB tumors, GRP4 = Group 4 MB tumors.

Analysis of the composite HCMV-associated late-gene signature (*UL76–UL88–UL99*) demonstrated significant subgroup differences (*p* = 0.005), with higher signal enrichment observed in Group 3 tumors compared with SHH tumors (adjusted *p* = 0.011) (Figure 3).

#### 3.3.2. WGS Cohort: Survival Analysis

Subgroup analyses showed that Group 3 tumors with elevated HCMV-associated late-gene signal (“HCMV-high Group 3”) had shorter progression-free survival (PFS; 44 vs. 76 months, *p* = 0.011) and overall survival (OS; 43 vs. 78 months, *p* = 0.002) than the remaining tumors. In contrast, stratification based solely on the composite HCMV-associated late-gene signature (composite Z-score ≥ median) was not significantly associated with survival outcomes across the entire cohort.

Exploratory multivariable Cox proportional hazards analyses showed that HCMV-high Group 3 status remained independently associated with poorer PFS (HR = 5.66, *p* = 0.029), event-free survival (EFS; HR = 7.59, *p* = 0.015), and OS (HR = 9.15, *p* = 0.014). Age and sex were not significantly associated with survival outcomes in these models (Table 1). Given the limited cohort size and the number of survival events, these findings should be interpreted with caution and considered hypothesis-generating. Nevertheless, the data support a potential association between the HCMV-associated molecular signal and adverse clinical outcomes in a subset of Group 3 medulloblastomas.

### 3.4. Multi-Omics Integration

A total of 28 pediatric medulloblastoma cases with matched tumor whole-genome sequencing (WGS), tumor RNA-seq, and germline blood WGS datasets were included in the integrative multi-omics analysis.

To evaluate concordance between genomic and transcriptomic HCMV-associated signals, each viral locus was classified according to combined RNA-seq and WGS-derived thresholds based on global 75th-percentile cutoffs (p75) for mean log_2_(TPM + 1) and mean log_2_(NVA + 1) values. Loci were categorized as RNA+/DNA+ when both transcriptomic and genomic signals exceeded the threshold, RNA+/DNA− when only RNA-seq signal was elevated, RNA−/DNA+ when only WGS signal was elevated, and RNA−/DNA− when neither signal exceeded the cutoff.

Using this classification framework, 6 loci were categorized as RNA+/DNA+, 7 as RNA+/DNA−, 7 as RNA−/DNA+, and 31 as RNA−/DNA−. Among the recurrently detected loci, *UL76*, *UL88*, and *UL99* showed the most consistent combined enrichment across RNA-seq and WGS datasets, particularly in RNA+/DNA+ samples, supporting a concordant low-level HCMV-associated signal across genomic and transcriptomic layers (Supplementary Table S3).

Overall, integrative analyses demonstrated heterogeneous patterns of HCMV-associated signal across tumors. While a subset of loci showed concordant genomic and transcriptomic enrichment, other loci were detected predominantly at the DNA level without corresponding transcriptomic signal. Group 4 tumors exhibited the highest number of RNA+/DNA+ loci, followed by Group 3 and SHH tumors, whereas WNT tumors showed the lowest levels of detectable HCMV-associated signal (Supplementary Table S3).

#### 3.4.1. Subgroup-Specific HCMV Late Signature

**Figure 4:**
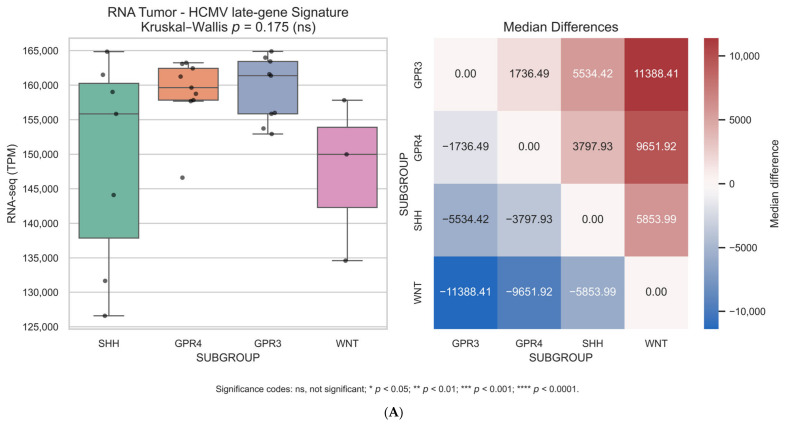

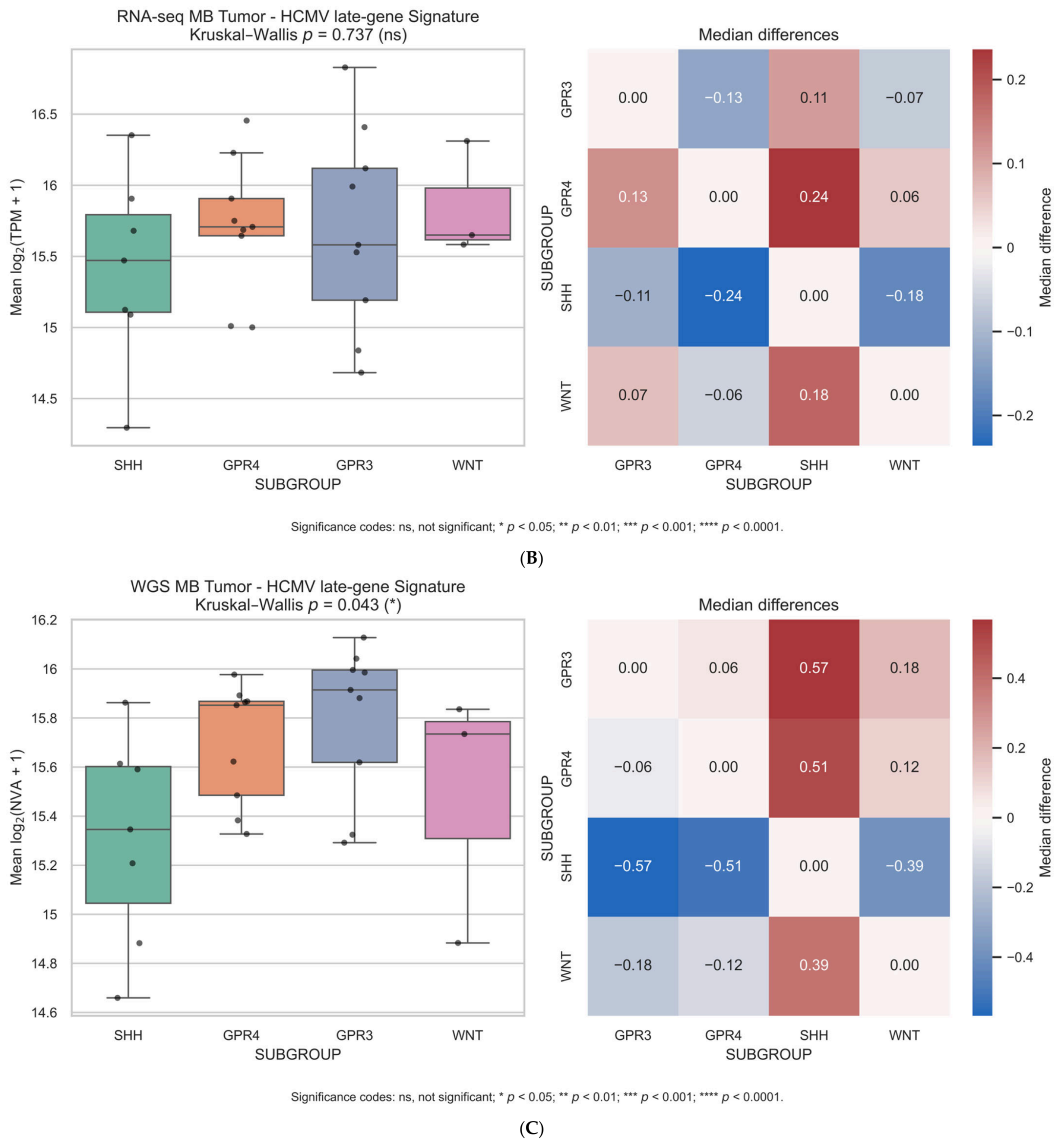

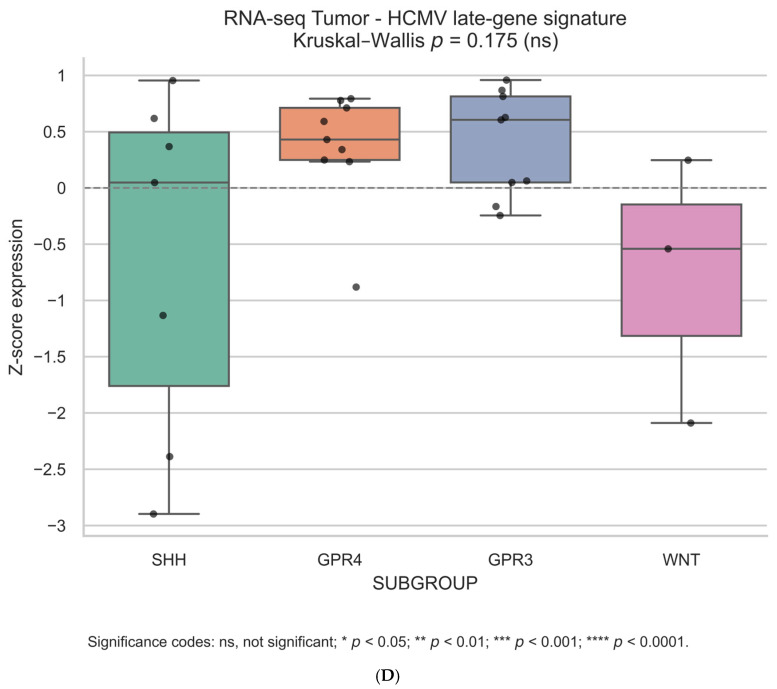

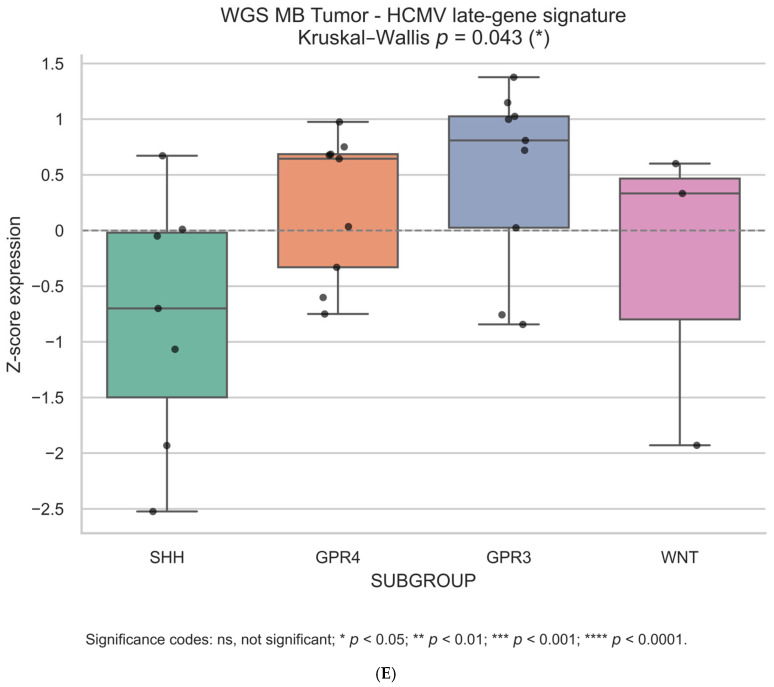
Integrated multi-omics analysis of the HCMV-associated late-gene signature across medulloblastoma subgroups. Integrated transcriptomic and genomic analyses were performed on 28 medulloblastoma cases with matched RNA-seq and WGS datasets. (**A**,**B**) RNA-seq analyses showed a trend toward increased expression of the HCMV-associated late-gene signature (*UL76*, *UL88*, *UL99*) in Group 3 tumors compared with other molecular subgroups, as indicated by both TPM and log_2_-transformed TPM values. However, these differences did not remain statistically significant after multiple-testing correction (Kruskal–Wallis *p* = 0.071; adjusted *p* = 0.428), consistent with the low abundance and variable distribution of transcript-level aligned reads across samples. (**C**) In contrast, WGS-derived analyses demonstrated significant subgroup-associated differences in HCMV-associated genomic signal (Kruskal–Wallis *p* = 0.043), with Group 3 tumors showing a higher composite late-gene signal than SHH tumors (mean ranks: 19.33 vs. 8.00; adjusted *p* = 0.038). (**D**,**E**) Z-score-normalized analyses integrating RNA-seq and WGS-derived features demonstrated concordant subgroup-associated patterns across molecular datasets, supporting the reproducible enrichment of low-level HCMV-associated molecular signals, particularly within Group 3 medulloblastomas. The integrated multi-omics analyses support heterogeneous but recurrent subgroup-associated HCMV-aligned molecular signatures rather than evidence of high-level or uniform viral abundance across tumors. GRP3 = Group 3 MB tumors, GRP4 = Group 4 MB tumors.

Analysis of the composite HCMV-associated late-gene signature (*UL76*, *UL88*, *UL99*) demonstrated significant subgroup-associated differences in the WGS dataset (*p* = 0.043). Group 3 tumors exhibited a higher HCMV-associated genomic signal compared with SHH tumors (adjusted *p* = 0.038) (Figure 4).

RNA-seq analyses showed a similar trend toward increased HCMV-associated late-gene signal in Group 3 tumors; however, these differences did not remain statistically significant after multiple-testing correction, likely reflecting increased variability and lower abundance of transcript-level aligned reads.

Together, these findings support heterogeneous but reproducible subgroup-associated enrichment of low-level HCMV-associated molecular signals, with the strongest relative enrichment observed in Group 3 medulloblastomas.

RNA-seq analyses demonstrated a similar trend toward increased HCMV-associated late-gene signal in Group 3 tumors; however, these differences were not statistically significant after correction for multiple testing. This likely reflects the low abundance and heterogeneous distribution of HCMV-aligned transcripts across samples, as well as the limited cohort size.

**Figure 5:**
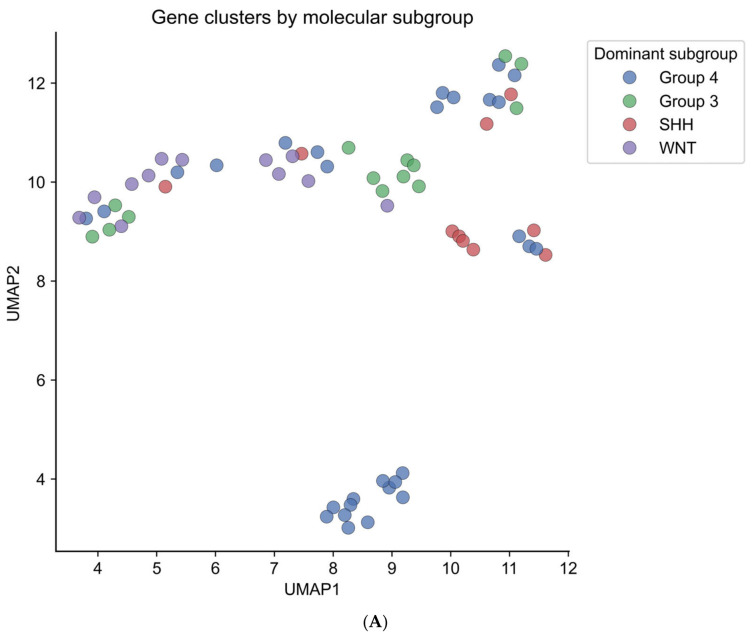

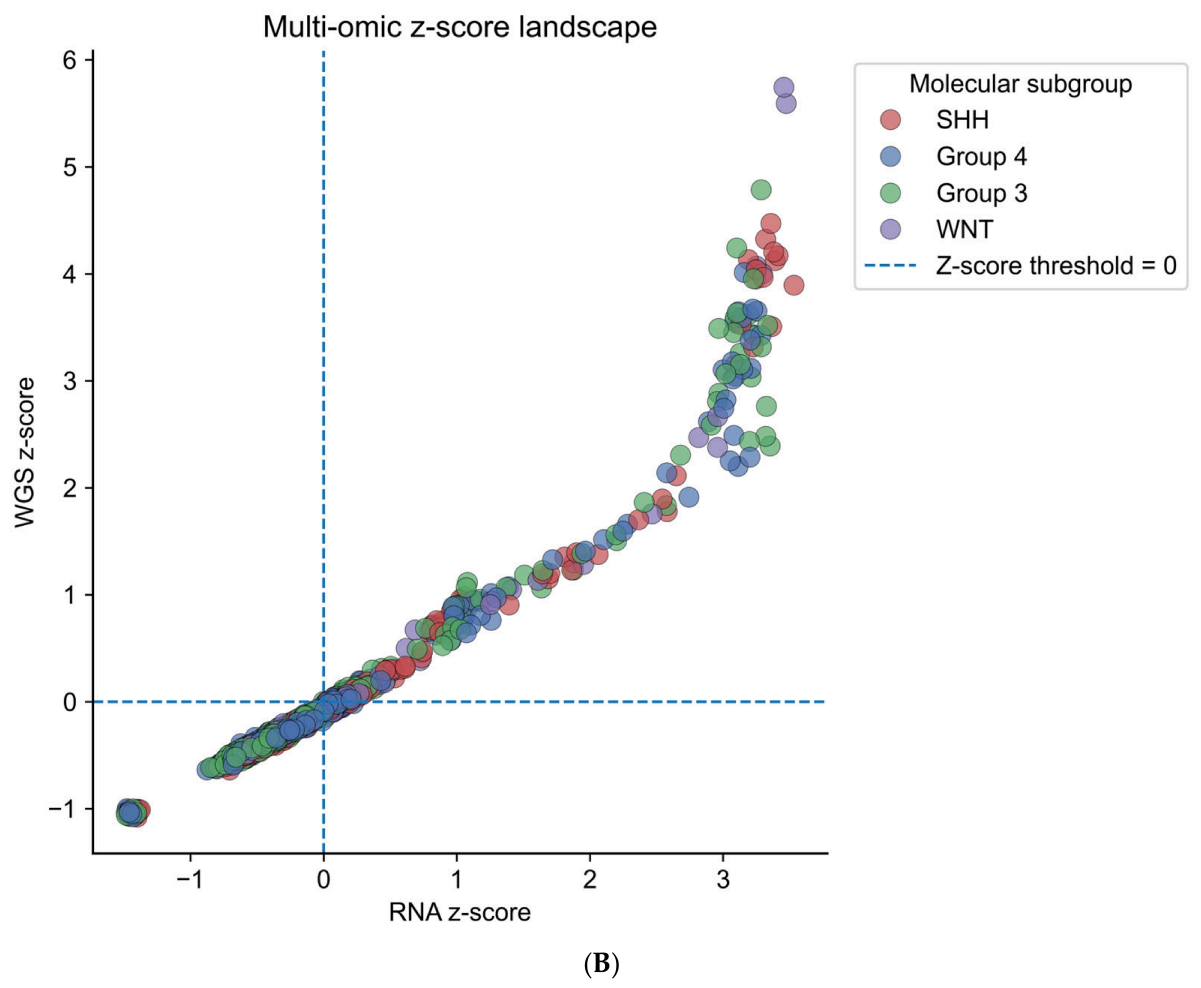
Integrated transcriptomic and genomic characterization of HCMV-associated molecular patterns in medulloblastoma. (**A**) Uniform Manifold Approximation and Projection (UMAP) analysis of integrated gene-level transcriptomic profiles demonstrates subgroup-associated clustering patterns across SHH, WNT, Group 3, and Group 4 medulloblastomas, highlighting distinct molecular architectures among tumor subgroups. (**B**) Integrated RNA-seq and WGS analyses using standardized Z-score normalization revealed heterogeneous relationships between transcriptomic and genomic HCMV-associated signals across tumors. A subset of loci showed elevated RNA expression without corresponding genomic enrichment (RNA+/DNA−), suggesting that low-level HCMV-associated transcriptional activity may occur independently of detectable DNA-level signal. Conversely, other loci showed concordant RNA+/DNA+ patterns, supporting reproducible detection of HCMV-associated molecular features across independent sequencing platforms. These analyses demonstrate heterogeneous yet recurrent subgroup-associated HCMV-aligned molecular patterns, particularly in Group 3 tumors, while also underscoring the low abundance and variable nature of the detected viral-associated signals.

Overall, the integrated analyses support the presence of heterogeneous but reproducible low-level HCMV-associated signals across medulloblastoma subgroups, with the strongest relative molecular enrichment observed in Group 3 tumors. Importantly, concordant patterns identified across independent genomic (WGS) and transcriptomic (RNA-seq) platforms reduce the likelihood that the observed signals are attributable solely to random alignment artifacts or background noise. In addition, a latent viral-associated signal score derived from principal component analysis (PCA) integrating WGS- and RNA-seq-derived features further supported these subgroup-associated patterns (Figure 5).

#### 3.4.2. Multi-Omics Integration: Survival Analysis

Within the integrated multi-omics cohort, Group 3 tumors with elevated HCMV-associated late-gene signal (“HCMV-high Group 3”) were associated with significantly reduced progression-free survival (PFS; 45 vs. 72 months, *p* = 0.004) and overall survival (OS; 45 vs. 69 months, *p* = 0.034). In contrast, stratification based solely on the HCMV-associated signal, independent of molecular subgroup, did not show significant associations with survival outcomes in Kaplan–Meier analyses.

Although limited by the small cohort size and low number of survival events, these findings support a potential association between combined Group 3 biology and elevated HCMV-associated molecular signal with adverse clinical outcome.

### 3.5. Combined Cohort Survival Analysis

Because of the limited sample size and relatively small number of survival events, all survival analyses should be interpreted as exploratory and hypothesis-generating.

To increase analytical power, we combined our previously reported Tübingen pediatric medulloblastoma cohort assessed by immunohistochemistry (IHC; *n* = 45) with the present WGS cohort (*n* = 39), resulting in a combined cohort of 84 cases. In the Tübingen cohort, HCMV late-antigen expression was evaluated using an anti-cytomegalovirus monoclonal antibody, clone 1G5.2 (MAB8127, Chemicon ®, Merck Millipore, Darmstadt, Germany), which recognizes late-antigen structural proteins, including pp28 (*UL99*). Cases with ≥25% positive tumor cells were classified as HCMV-high [1].

In the WGS cohort, HCMV-high status was defined using the composite HCMV-associated late-gene signature derived from *UL76*, *UL88*, and *UL99* loci, with values above the cohort median composite Z-score classified as high signal.

Although the IHC- and WGS-based approaches represent different biological and technical readouts, both were designed to capture complementary HCMV-associated molecular features at the protein and genomic levels. Each cohort was therefore dichotomized according to its predefined HCMV-high threshold for exploratory integrative survival analyses.

The combined cohort included WNT (*n* = 13, 16%), SHH (*n* = 19, 23%), Group 3 (*n* = 23, 27%), and Group 4 tumors (*n* = 29, 34%).

Across the entire cohort, Group 3 tumors demonstrated poorer clinical outcomes compared with the remaining molecular subgroups, including shorter PFS (median 19 vs. 76 months, *p* = 0.118), EFS (median 94 vs. 147 months, *p* = 0.070), and significantly reduced OS (mean 83 vs. 141 months, *p* = 0.013). No significant survival differences were observed for the remaining molecular subgroups (Supplementary Table S4).

**Figure 6:**
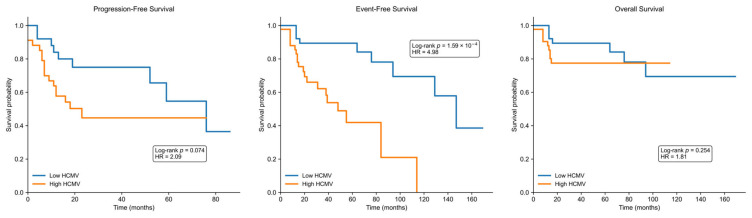

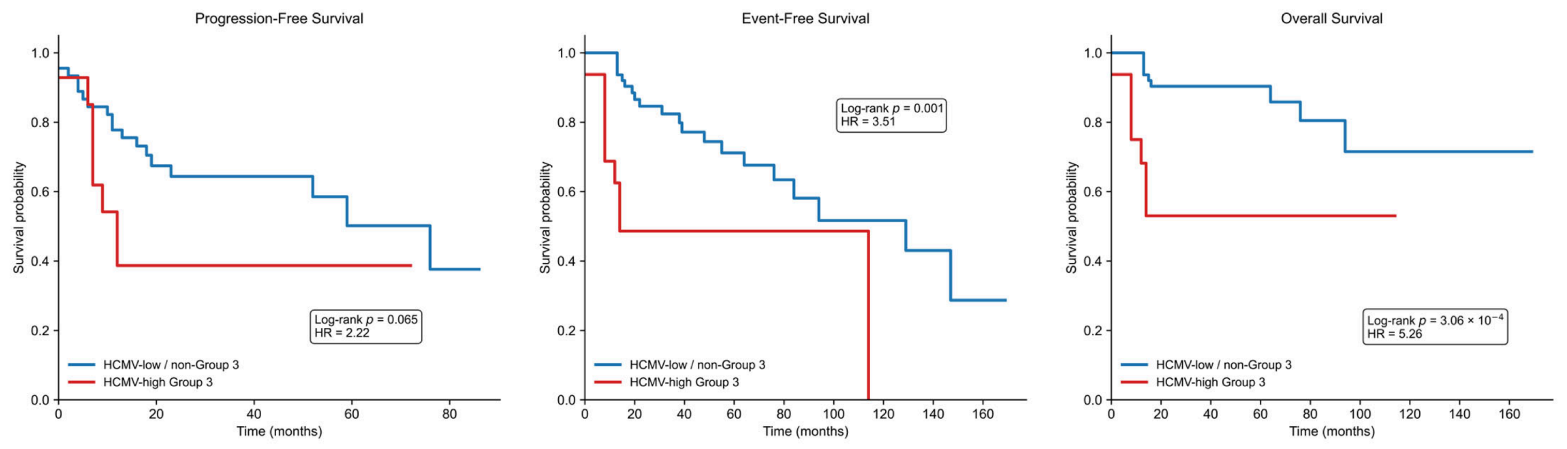
Exploratory Kaplan–Meier survival analysis of the combined pediatric medulloblastoma cohort (*n* = 84), integrating whole-genome sequencing (WGS; *n* = 39) and immunohistochemistry (IHC; *n* = 45) datasets. (**Upper panel**) Cases with high HCMV-associated signal, defined by cohort-specific WGS or IHC criteria, demonstrated significantly reduced event-free survival (EFS; median 55 vs. 147 months, log-rank *p* < 0.001) and overall survival (OS; mean 90 vs. 134 months, *p* = 0.034), whereas progression-free survival (PFS) showed a non-significant trend toward poorer outcome (median 23 vs. 76 months, *p* = 0.074). (**Lower panel**) Subgroup analysis revealed that HCMV-high Group 3 tumors were associated with significantly worse EFS (mean 14 vs. 129 months, *p* = 0.001) and OS (mean 64 vs. 137 months, *p* < 0.001), while the difference in PFS did not reach statistical significance (mean 12 vs. 76 months, *p* = 0.065).

Kaplan–Meier analyses demonstrated that tumors with high HCMV-associated signal (WGS/IHC combined) were associated with significantly reduced event-free survival (EFS; median 55 vs. 147 months, *p* < 0.001). A non-significant trend toward shorter progression-free survival was observed (PFS; median 23 vs. 76 months, *p* = 0.074), whereas overall survival was significantly reduced in the high-signal group (OS; mean 90 vs. 134 months, *p* = 0.034) (Figure 6, upper panel).

Subgroup analyses further demonstrated that HCMV-high Group 3 tumors were associated with markedly reduced EFS (mean 14 vs. 129 months, *p* = 0.001) and OS (mean 64 vs. 137 months, *p* < 0.001). Although PFS differences did not reach statistical significance (mean 12 vs. 76 months, *p* = 0.065), a clear trend toward poorer outcome was observed (Figure 6, lower panel). No significant associations with survival were identified for the remaining molecular subgroups (Supplementary Table S5; Supplementary Figure S1).

Independent analyses restricted to Group 3 tumors (*n* = 23) similarly demonstrated poorer outcomes among tumors with elevated HCMV-associated late-gene signal, including significantly reduced EFS (mean 64 vs. 117 months; median 114 vs. 129 months; *p* = 0.042). Trends toward shorter PFS (median 12 vs. 52 months; log-rank *p* = 0.309) and reduced OS (mean 64 vs. 117 months; *p* = 0.105) were also observed, although these did not reach statistical significance.

#### Multivariate Analysis

Exploratory multivariable Cox proportional hazards models were constructed for the combined cohort (*n* = 84) using dichotomized clinical and molecular variables, including age (<3 vs. ≥3 years), sex, metastatic status (M0 vs. M1–M3), extent of resection (STR vs. GTR), molecular subgroup (Group 3 vs. others), and HCMV-associated signal (high vs. low). Because of the limited number of survival events compared with the number of covariates, hazard ratio estimates should be interpreted with caution.

**Figure 7:**
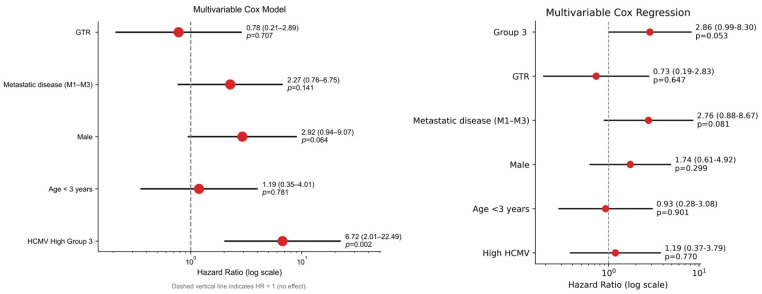
Exploratory multivariable Cox regression analysis identifying a high-risk HCMV-associated subgroup in pediatric medulloblastoma (*n* = 84). Forest plots summarize hazard ratios (HRs) and 95% confidence intervals (CIs) derived from multivariable Cox proportional hazards models evaluating progression-free survival (PFS), event-free survival (EFS), and overall survival (OS). Models including individual clinicopathological variables (**right panel**) identified age < 3 years, metastatic disease, and high HCMV-associated signal as independent predictors of adverse outcome, while Group 3 tumors were associated with reduced OS (HR = 2.84, *p* = 0.050). Models incorporating the combined variable “HCMV-high Group 3 tumors” (**left panel**) demonstrated the strongest association with poor clinical outcome, including reduced OS (HR = 6.43, *p* = 0.002) and EFS (HR = 3.50, *p* = 0.016), supporting the presence of a biologically high-risk subgroup characterized by combined Group 3 biology and elevated HCMV-associated signal.

Age younger than 3 years was independently associated with poorer progression-free survival (PFS; HR = 3.19, *p* = 0.017). Metastatic disease (HR = 3.42, *p* = 0.008) and elevated HCMV-associated signal (HR = 3.86, *p* = 0.010) were independently associated with reduced event-free survival (EFS). Group 3 molecular subgroup status was associated with poorer overall survival (OS; HR = 2.84, *p* = 0.050) (Figure 7, right panel).

Importantly, the combined variable “HCMV-high Group 3” demonstrated the strongest association with adverse clinical outcome, remaining independently associated with reduced OS (HR = 6.43, *p* = 0.002) and EFS (HR = 3.50, *p* = 0.016), together with metastatic disease (HR = 4.51, *p* < 0.001) (Figure 7, left panel).

**Figure 8:**
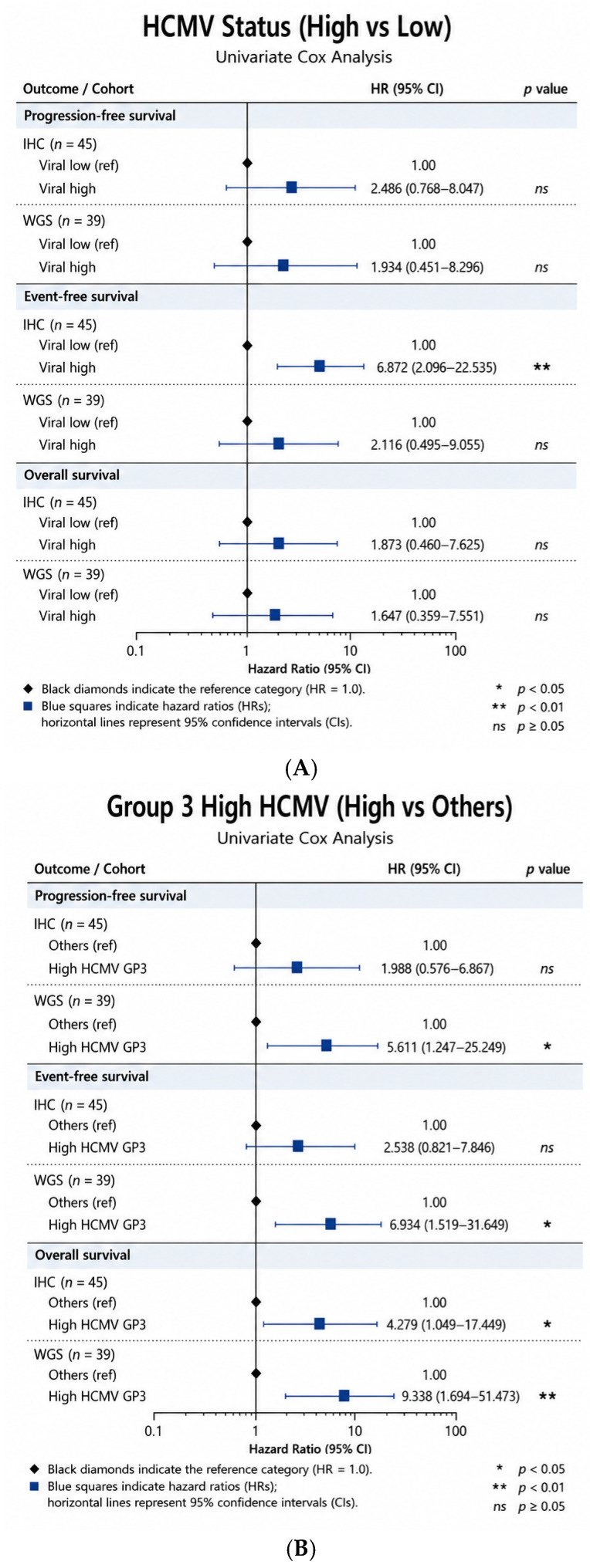
Exploratory univariate Cox proportional hazards analyses of HCMV-associated signal and HCMV-high Group 3 tumors in pediatric medulloblastoma. Forest plots display hazard ratios (HRs) with 95% confidence intervals (CIs) for progression-free survival (PFS), event-free survival (EFS), and overall survival (OS) derived from univariate Cox regression models in the immunohistochemistry (IHC; *n* = 45) and whole-genome sequencing (WGS; *n* = 39) cohorts. (**A**) compares cases with high versus low HCMV-associated signal, while (**B**) evaluates the combined subgroup “HCMV-high Group 3 tumors” versus all remaining tumors. Squares indicate HR estimates and horizontal lines represent 95% CIs; the vertical reference line indicates HR = 1. Significant associations (* *p* < 0.05, ** *p* < 0.01) are shown in bold. HCMV-associated signal detected by IHC showed a significant association with reduced EFS (**A**), whereas the HCMV-high Group 3 subgroup demonstrated more consistent associations with PFS, EFS, and OS across both cohorts (**B**), supporting enrichment for adverse clinical outcomes within this biologically high-risk subgroup.

Univariable forest plot analyses demonstrated that HCMV status assessed by immunohistochemistry was significantly associated with reduced EFS (HR = 6.8, *p* = 0.001). In contrast, the combined HCMV-high Group 3 subgroup showed consistent associations with PFS, EFS, and OS across both the IHC and WGS cohorts (Figure 8).

Additional multivariable analyses of the combined cohort showed that metastatic disease (HR = 3.4, *p* = 0.006) and HCMV-high Group 3 status (HR = 7.3, *p* = 0.001) were independently associated with poorer EFS. Furthermore, HCMV-high Group 3 status was the only independent predictor associated with worse OS (HR = 4.5, *p* = 0.018) (Table 2).

### 3.6. Orthogonal Validation of HCMV-Associated Signal and Genomic Correlates in Group 3 Medulloblastoma

To further evaluate the biological context and reproducibility of the observed HCMV-associated signal, an orthogonal analysis integrating RNA-seq and whole-genome sequencing (WGS) data was performed in Group 3 medulloblastoma tumors.

WGS analyses revealed heterogeneous genomic alterations characteristic of aggressive Group 3 biology, including variable *MYC* amplification, *TP53*-associated coverage imbalance, and chromosome 17 alterations, together with detectable low-level HCMV-associated genomic signal.

Samples GP3_9 and GP3_10 demonstrated the highest *MYC* amplification, with *MYC*/mean-depth ratios of 14.71 and 34.02, respectively. In contrast, GP3_5 and GP3_8 showed reduced *TP53* coverage and elevated chr17q/chr17p ratios, consistent with partial 17p loss and relative 17q gain. Whereas most tumors showed relatively balanced chr17q/chr17p ratios (1.0–1.1), GP3_5 and GP3_10 exhibited ratios >2.3, suggesting increased chromosomal instability.

Notably, elevated HCMV-associated late-gene signature scores [log_2_(NVA + 1)] co-occurred with increased *MYC*-associated genomic signal and chr17q gain, suggesting preferential enrichment of HCMV-associated molecular features within genomically unstable Group 3 tumors.

To further validate viral detection, unmapped WGS reads were reanalyzed against the HCMV reference genome following stringent MAPQ > 20 filtering. Low-abundance HCMV-aligned reads were reproducibly identified across multiple Group 3 tumors, with recurrent enrichment involving *UL88*, *UL76*, and *UL99* loci. Among these, *UL88* demonstrated the strongest relative enrichment, including the highest RPM values, mean depth coverage, and detectable breadth across multiple sequencing thresholds, despite the extremely low overall abundance of viral-aligned reads.

RNA-seq analyses using a combined human (hg19) and HCMV reference genome further supported concordant detection of rare HCMV-associated transcripts within a subset of Group 3 tumors. After stringent filtering, HCMV-aligned transcripts were reproducibly identified in four tumors and predominantly mapped to the same late-gene regions identified in WGS analyses.

Although HCMV-associated transcript abundance accounted for only a minute fraction of total mapped reads (~10^−6^ CPM), tumors with detectable HCMV-associated transcriptomic signal frequently exhibited increased *MYC* expression and greater genomic imbalance. In particular, GP3_3 and GP3_9 exhibited elevated *MYC* expression and increased *MYC/TP53* expression ratios, whereas tumors lacking detectable HCMV-associated aligned reads generally demonstrated lower *MYC* expression and reduced chromosomal imbalance.

**Figure 9:**
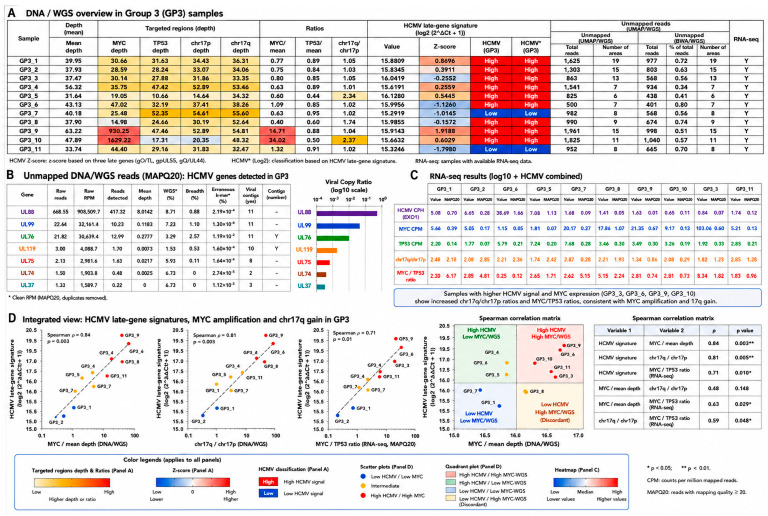
Integrated multi-omics characterization and orthogonal validation of HCMV-associated molecular signals in Group 3 medulloblastoma. (**A**) Genomic overview of Group 3 (GP3) medulloblastoma samples from whole-genome sequencing (WGS), including mean sequencing depth, *MYC* and *TP53* coverage, chromosome 17p/17q depth ratios, and the composite HCMV late-gene signature [*UL76–UL88–UL99*; log_2_(NVA + 1)]. Samples were stratified into HCMV-high and HCMV-low groups based on composite Z-scores. The number of unmapped HCMV-aligned reads before and after MAPQ ≥ 20 filtering, as well as RNA-seq availability, is also shown. (**B**) Orthogonal analysis of unmapped WGS reads realigned to the HCMV reference genome (NC_006273.2). Recurrent enrichment was observed at the late-gene loci *UL76*, *UL88*, and *UL99*, which exhibited the highest RPM values, genomic coverage, and prevalence across Group 3 tumors, supporting a reproducible, low-level HCMV-associated genomic signal. (**C**) RNA-seq analysis using a combined hg19 + HCMV reference genome demonstrated concordant detection of low-abundance HCMV-associated transcripts in a subset of Group 3 tumors. Comparative analyses of HCMV CPM, *MYC* CPM, *TP53* CPM, chr17q/chr17p ratios, and *MYC/TP53* expression ratios revealed increased *MYC*-associated activity and chromosomal imbalance in tumors with elevated HCMV-associated transcriptional signal. (**D**) Integrated correlation analyses showed positive associations between the HCMV late-gene signature and molecular features of aggressive Group 3 biology, including *MYC* amplification, chr17q gain, and elevated *MYC/TP53*-associated ratios. Spearman correlation analysis further supported concordance between HCMV-associated genomic and transcriptomic signals and markers of genomic instability. These findings support the presence of reproducible, low-level HCMV-associated molecular signatures that are preferentially enriched in biologically high-risk Group 3 medulloblastoma.

Collectively, these orthogonal analyses demonstrate reproducible low-level HCMV-associated genomic and transcriptomic signals across independent sequencing platforms in a subset of Group 3 medulloblastomas. The concordance between WGS and RNA-seq findings supports the robustness of the observed molecular signal and underscores its extremely low abundance. These findings are therefore more consistent with restricted or low-level HCMV-associated transcriptional activity rather than evidence of productive viral infection (Figure 9).

## 4. Discussion

In this study, we performed an integrated multi-omics analysis demonstrating that low-level HCMV-associated genomic and transcriptomic signals are recurrently detectable in pediatric medulloblastoma, particularly within Group 3 tumors, and are associated with adverse clinical outcomes. By combining WGS and RNA-seq, orthogonal genomic validation analyses, and survival data from independent pediatric cohorts, we identified a reproducible HCMV-associated late-gene signature composed of *UL76*, *UL88*, and *UL99* that was enriched in biologically aggressive tumors and associated with reduced survival [10,11].

Importantly, the current study extends our previous immunohistochemical findings linking HCMV late-antigen expression with poor prognosis in pediatric medulloblastoma and supports the presence of a reproducible molecular HCMV-associated signal across independent genomic and transcriptomic platforms [1]. However, the extremely low abundance of viral-aligned reads observed in our datasets indicates that these findings should be interpreted with caution and are more consistent with restricted or low-level viral-associated transcriptional activity than with evidence of productive viral infection.

### 4.1. Principal Findings and Biological Interpretation

The HCMV-associated late-gene signature (*UL76–UL88–UL99*) represented the most recurrent and reproducible viral-associated pattern identified across RNA-seq and WGS datasets. This signature was preferentially enriched in Group 3 tumors and showed the strongest survival associations within the HCMV-high Group 3 subgroup, suggesting a potential interaction between aggressive tumor biology and HCMV-associated molecular activity.

The orthogonal validation further supported these findings by demonstrating concordant detection of low-level HCMV-associated genomic and transcriptomic signals across independent sequencing modalities. Tumors with elevated HCMV-associated signal frequently exhibited *MYC* amplification, chromosome 17q imbalance, and increased *MYC/TP53* ratios, suggesting enrichment of viral-associated molecular activity in genomically unstable Group 3 tumors [12,13].

Mechanistically, HCMV has been implicated in multiple pathways relevant to tumor progression, including inflammation, immune modulation, angiogenesis, genomic instability, and cellular stress responses. *UL76* has been associated with activation of DNA damage responses, NF-κB signaling, IL-8 production, and chromosomal instability, all of which may contribute to tumor-supportive inflammatory signaling [3,7]. *UL88* has been reported to interfere with innate immune signaling by degrading MyD88, thereby facilitating immune evasion and the persistence of viral-associated molecular activity [6]. *UL99*, although primarily a structural late protein, may reflect persistent late-stage viral-associated transcriptional activity within tumor-associated microenvironments [4,8].

An additional mechanism potentially relevant to tumor immune evasion is the expression of viral interleukin-10 homologs (cmvIL-10) by HCMV. HCMV-derived cmvIL-10 has been shown to suppress anti-tumor immune responses, inhibit antigen presentation, and modulate major histocompatibility complex (MHC) expression, thereby contributing to an immunologically permissive microenvironment. Through these mechanisms, infected or HCMV-associated cells may maintain a relatively “normal” MHC phenotype and evade immune recognition despite altered tumor-associated biology. This immunomodulatory effect may be particularly relevant in medulloblastoma, where immune surveillance mechanisms already appear limited within the tumor microenvironment [5].

Collectively, these findings support the concept that HCMV may function as an oncomodulatory rather than directly oncogenic virus in medulloblastoma, potentially influencing tumor progression indirectly through immune modulation, inflammatory signaling, and enhancement of tumor-supportive cellular pathways [3,5,11,14].

### 4.2. HCMV-Associated Molecular Signal in Group 3 Medulloblastoma

Our findings consistently demonstrated preferential enrichment of HCMV-associated signal within Group 3 medulloblastoma, a subgroup characterized by poor prognosis, frequent molecular metastasis, *MYC* activation, chromosomal instability, and substantial biological heterogeneity [2,9].

The observed overlap between elevated HCMV-associated signal, *MYC* dysregulation, and chromosome 17 imbalance suggests that aggressive tumor-associated molecular states may facilitate or coexist with low-level HCMV-associated transcriptional activity [15,16]. Conversely, HCMV-associated inflammatory and immune-modulatory pathways may further contribute to tumor progression by enhancing oncogenic signaling and impairing anti-tumor immune responses [12,13].

Importantly, the current data do not establish causality between HCMV-associated signal and medulloblastoma biology. Rather, the findings support an association between HCMV-aligned molecular activity and biologically aggressive Group 3 tumors. Whether these signals reflect direct viral activity, tumor-associated permissiveness, inflammatory microenvironmental states, or shared transcriptional programs remains unresolved and will require additional mechanistic investigation.

### 4.3. HCMV as a Potential Oncomodulatory Factor

The role of HCMV in human malignancies remains controversial. Unlike classical oncogenic viruses, HCMV is not generally considered a transforming virus. Instead, growing evidence supports an oncomodulatory role in which HCMV-associated molecular activity may influence tumor progression by modulating inflammation, angiogenesis, immune evasion, and cellular signaling pathways [5,11,14].

Our findings contribute to this ongoing discussion [1,11,12,13] by demonstrating reproducible, low-level HCMV-associated genomic and transcriptomic signals in pediatric medulloblastoma that correlate with clinically relevant outcomes, particularly in Group 3 tumors. Concordance among RNA-seq, WGS, orthogonal analyses, and prior immunohistochemical findings strengthens the biological consistency of the observed signal, although definitive evidence of active viral infection remains lacking.

Importantly, the extremely low abundance of viral-aligned reads underscores the need for cautious interpretation. These findings should therefore be viewed as evidence of recurrent HCMV-associated molecular signatures rather than definitive proof of productive HCMV infection within tumor cells.

### 4.4. Multi-Omics Integration and Analytical Robustness

A major strength of this study is the integration of multiple analytical independent layers, including genomic, transcriptomic, orthogonal validation, immunohistochemical, and survival data. The reproducible detection of HCMV-associated signal across WGS and RNA-seq platforms supports the robustness of the observed molecular patterns and reduces the likelihood that findings are solely attributable to technical artifacts.

The classification of viral-associated loci into RNA+/DNA+, RNA+/DNA−, RNA−/DNA+, and RNA−/DNA− categories further demonstrated substantial heterogeneity across tumors, consistent with known HCMV biology, including latency-like states, intermittent transcriptional activity, and restricted viral expression programs [3,5].

Importantly, our orthogonal validation strengthens confidence in the reproducibility of the observed HCMV-associated signal by demonstrating concordant enrichment of *UL76*, *UL88*, and *UL99* across independent sequencing strategies after stringent filtering.

Nevertheless, technical limitations remain highly relevant in studies investigating low-abundance viral-associated signals. The possibility of background alignment noise, contamination, latent viral DNA fragments, or alignment artifacts cannot be completely excluded despite stringent MAPQ filtering and the inclusion of multiple control datasets.

### 4.5. Clinical Implications

The current findings suggest that HCMV-associated molecular signatures may provide additional prognostic information in pediatric medulloblastoma, particularly within Group 3 tumors. Across independent analyses, HCMV-high Group 3 tumors consistently demonstrated poorer survival outcomes compared with other molecular subgroups.

Although these findings remain exploratory, they suggest that virus-associated inflammatory or immunomodulatory pathways may contribute to biologically aggressive tumor behavior in selected patients.

Importantly, the present study does not support immediate clinical application of antiviral therapy in medulloblastoma. However, prior studies in glioblastoma and other malignancies have explored antiviral and immunomodulatory approaches targeting HCMV-associated pathways, although these remain controversial and incompletely validated [11,17,18]. Future translational studies will therefore require rigorous mechanistic validation before any therapeutic implications can be considered in pediatric medulloblastoma.

### 4.6. Technical Considerations and Limitations

This study has several important limitations. First, the sample size, particularly for the integrated multi-omics analyses, remains relatively small, limiting statistical power and increasing uncertainty around hazard ratio estimates. Second, sequencing-based viral detection at extremely low abundance levels remains technically challenging. Although stringent alignment filtering, orthogonal analyses, and multiple control datasets were incorporated to improve specificity, low-level viral-aligned reads cannot definitely distinguish between latent viral fragments, restricted transcriptional states, contamination, or biologically relevant viral-associated activity. Third, bulk sequencing approaches do not permit identification of the precise cellular origin of virus-associated transcripts, which may arise from tumor cells, infiltrating immune cells, stromal components, or other microenvironmental sources.

Fourth, different methodologies were used across cohorts (IHC vs. WGS-derived viral signatures). Although both approaches targeted HCMV-associated late-gene activity, they represent distinct biological readouts and are not directly interchangeable.

Finally, additional validation using optimized viral detection methodologies will be essential. Future studies should incorporate optimized PCR/DNA-based approaches specifically designed for low-level HCMV detection, similar to previously described optimized immunohistochemical retrieval protocols such as the enhanced antigen-retrieval techniques reported by Cobbs and colleagues for HCMV detection in tumor tissues [19,20]. Standardized optimization of PCR sensitivity, viral enrichment methods, and orthogonal validation workflows will likely be critical to clarify the biological significance of these low-abundance HCMV-associated signals.

### 4.7. Future Directions

Future studies should validate the HCMV-associated late-gene signature in larger independent cohorts using standardized, multi-platform methodologies that integrate optimized PCR/DNA detection, transcriptomics, immunohistochemistry, and spatially resolved analyses. Single-cell RNA sequencing and spatial transcriptomics will be particularly important for determining the precise cellular origin and spatial distribution of HCMV-associated molecular signals in medulloblastoma tissues.

Additional mechanistic studies are needed to clarify whether *UL76*, *UL88*, *UL99*, or HCMV-derived immunomodulatory proteins, such as cmvIL-10, contribute directly to tumor-associated immunosuppression, *MYC* dysregulation, genomic instability, or treatment resistance.

Understanding host–virus interaction networks in high-risk Group 3 tumors may ultimately identify novel biomarkers or therapeutic vulnerabilities relevant to the biology of aggressive pediatric medulloblastoma.

## 5. Conclusions

In conclusion, this integrated multi-omics study identifies recurrent low-level HCMV-associated genomic and transcriptomic signals in pediatric medulloblastoma, particularly within Group 3 tumors, where elevated HCMV-associated late-gene signature activity correlated with adverse clinical outcomes. The reproducible enrichment of *UL76*, *UL88*, and *UL99* across independent RNA-seq, WGS, orthogonal validation, and immunohistochemical datasets supports the presence of a biologically consistent HCMV-associated signature in a subset of aggressive molecular medulloblastomas. However, the extremely low abundance of viral-aligned reads indicates that these findings should be interpreted cautiously and are most consistent with restricted or low-level HCMV-associated transcriptional activity rather than productive viral infection. Further validation using larger independent cohorts, optimized PCR/DNA detection strategies, standardized immunohistochemical methodologies, and mechanistic experimental studies will be essential to clarify the biological and clinical significance of HCMV-associated molecular activity in pediatric medulloblastoma.

## Figures and Tables

**Table 1:** Cox multivariable analysis in the WGS cohort (*n* = 39).

	Progression-Free Survival (PFS)					Event-Free Survival (EFS)				Overall Survival (OS)			
Variables		HR	95% CI Lower	95% CI Upper	*p*-Value	HR	95% CI Lower	95% CI Upper	*p*-Value	HR	95% CI Lower	95% CI Upper	*p*-Value
Age		0.993	0.819	1.205	0.946	0.994	0.819	1.206	0.951	0.982	0.796	1.211	0.865
Gender	Female	1			0.953	1			0.748	1			0.918
	Male	1.052	0.196	5.653		1.325	0.239	7.359		0.907	0.140	5.860	
HCMV-high Group 3	No	1			0.029 *	1			0.015 *	1			0.014 *
	Yes	5.665	1.200	26.745		7.596	1.475	39.116		9.158	1.573	53.319	

Progression-free survival (PFS) events (*n* = 8; censored *n* = 31); event-free survival (EFS) events (*n* = 8; censored *n* = 31); overall survival (OS) events (*n* = 7; censored *n* = 32). In multivariable Cox regression, the combined variable “HCMV-high Group 3” was an independent predictor of worse PFS, EFS, and OS (*p* < 0.05), whereas age (continuous) and sex (dichotomous) were not significant covariates. For this analysis, only variables present in all cases were included; metastatic status and extent of resection (EOR) were not available for the entire cohort. * Significant *p*-value < 0.05.

**Table 2:** Cox multivariable analysis considering the combined cohort (*n* = 84).

	Progression-Free Survival (PFS)					Event-Free Survival (EFS)				Overall Survival (OS)			
Variables		HR	95% CI Lower	95% CI Upper	*p*-Value	HR	95% CI Lower	95% CI Upper	*p*-Value	HR	95% CI Lower	95% CI Upper	*p*-Value
Age	under 3 yr	2.566	1.023	6.433	0.045 *	1.437	0.581	3.554	0.433	0.720	0.215	2.405	0.593
	over 3 yr	1				1				1			
Gender	Female	1				1				1			
	Male	1.429	0.555	3.679	0.459	2.533	1.079	5.945	0.033 *	2.778	0.898	8.590	0.076
Metastasis	M0	1				1				1			
	M1–M3	2.307	0.962	5.532	0.061	3.427	1.428	8.225	0.006 *	2.659	0.842	8.403	0.096
HCMV-high WNT	No	1				1				1			
	yes	1.302	0.144	11.793	0.814	1.668	0.193	14.392	0.642	0.000	0.000		0.989
HCMV-high SHH	No	1				1				1			
	yes	3.628	0.658	20.004	0.139	5.323	0.951	29.779	0.057	0.000	0.000		0.990
HCMV-high Group 3	No	1				1				1			
	yes	2.214	0.744	6.588	0.153	7.308	2.382	22.424	<0.001 *	4.552	1.290	16.061	0.018 *
HCMV-high Group 4	No	1				1				1			
	yes	1.832	0.610	5.498	0.281	2.951	0.894	9.736	0.076	0.444	0.079	2.494	0.356

Progression-free survival (PFS), event-free survival (EFS), and overall survival (OS) events. In multivariable Cox regression, the combined variable “HCMV-high Group 3” was an independent predictor of worse EFS and OS (*p* < 0.05). * Significant *p*-value < 0.05.

## Data Availability

The data supporting the findings of this study are available from the European Genome-phenome Archive (EGA) under controlled access in accordance with the International Cancer Genome Consortium (ICGC) data access policies. Access to these datasets is subject to approval by the respective data access committees. Publicly available control brain transcriptomic datasets used for comparative analyses are available from their original repositories as cited within the manuscript. All relevant processed data generated in this study are included within the article and its Supplementary Materials. Additional data are available from the corresponding author upon reasonable request.

## Supplementary Materials

The following supporting information can be downloaded at https://www.mdpi.com/article/10.3390/biomedicines14061328/s1: Supplementary Materials S1. HCMV late-gene expression signature across medulloblastoma molecular subgroups and control tissues. Supplementary Materials S2. Differential expression of the HCMV late-gene signature across medulloblastoma molecular subgroups. Figure S1. Survival outcomes of HCMV-high molecular medulloblastoma subgroups in the combined cohort (n = 84). Table S1. Comparison of HCMV-aligned RNA-seq reads across tumors, controls, and infected cell cultures. Table S2. HCMV transcripts detected in pediatric medulloblastoma tumors. Table S3. Multi-omics classification of HCMV-associated genes based on integrated RNA-seq and whole-genome sequencing analyses. Table S4. Survival outcomes according to medulloblastoma molecular subgroup in the combined cohort (n = 84). Table S5. Survival outcomes of HCMV-high molecular subgroups in the combined cohort (n = 84).

## Abbreviations

The following abbreviations are used in this manuscript:

Abbreviation	Definition
ANOVA	Analysis of variance
BAM	Binary alignment map
bp	Base pairs
CI	Confidence interval
cmvIL-10	Cytomegalovirus-encoded interleukin-10
CPM	Counts per million
DNA	Deoxyribonucleic acid
EFS	Event-free survival
EGA	European Genome-Phenome Archive
ENA	European Nucleotide Archive
EOR	Extent of resection
FASTQ	Sequencing read file format
GEO	Gene Expression Omnibus
GP3	Group 3 medulloblastoma
GRCh37 (hg19)	Human reference genome build 37
GTR	Gross total resection
HCMV	Human cytomegalovirus
HFF	Human foreskin fibroblast
HR	Hazard ratio
ICGC	International Cancer Genome Consortium
IHC	Immunohistochemistry
IL-8	Interleukin-8
KM	Kaplan–Meier
LCA	Large cell/anaplastic
LA	Late antigen
log_2_(NVA + 1)	Log_2_-transformed normalized viral abundance values
log_2_(TPM + 1)	Log_2_-transformed transcripts per million values
MAPQ	Mapping quality
MB	Medulloblastoma
MHC	Major histocompatibility complex
*MYC*	*MYC* proto-oncogene
MyD88	Myeloid differentiation primary response protein 88
NCBI	National Center for Biotechnology Information
NF-κB	Nuclear factor kappa B
NPC	Neural progenitor cell
NVA	Normalized viral abundance
OS	Overall survival
PCA	Principal component analysis
PCR	Polymerase chain reaction
PFS	Progression-free survival
pp28	Phosphoprotein 28 encoded by *UL99*
RNA	Ribonucleic acid
RNA-seq	RNA sequencing
RPM	Reads per million
SD	Standard deviation
SHH	Sonic hedgehog
snRNA-seq	Single-nucleus RNA sequencing
STR	Subtotal resection
*TP53*	Tumor protein p53 gene
TPM	Transcripts per million
*UL76/UL88/UL99*	HCMV late genes encoding viral proteins pUL76, pUL88, and pp28
UMAP	Uniform manifold approximation and projection
WGS	Whole-genome sequencing
WNT	Wingless/integrated signaling subgroup
Z-score	Standardized expression value
