# Supplementary material for: Integrated Multi-Omics Analysis Reveals an HCMV-Associated Late-Gene Signature Associated with Poor Survival in Pediatric Group 3 Medulloblastoma
Source: Biomedicines. 2026 Jun 11;14(6):1328. doi: 10.3390/biomedicines14061328 (PMC13296392; doi:10.3390/biomedicines14061328)

Supplementary material 1 - HCMV late-gene expression signature across medulloblastoma molecular subgroups and control tissues.

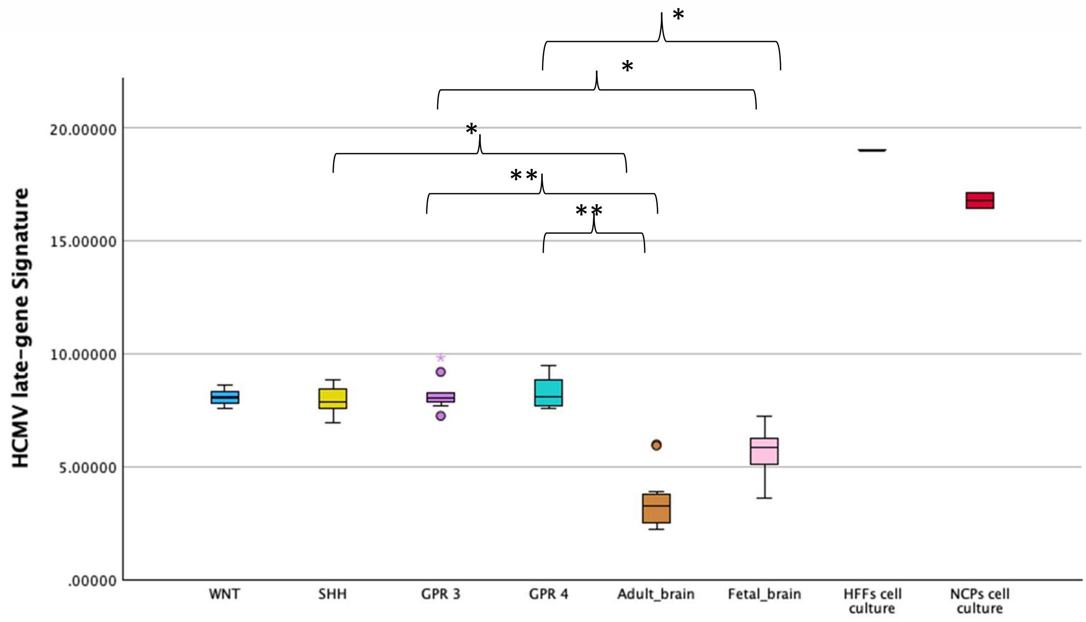

Kruskal–Wallis analysis of the composite HCMV late-gene signature (UL76, UL88, and UL99; $\log_2$(TPM + 1)) across WNT, SHH, Group 3, and Group 4 medulloblastomas, compared with adult brain, fetal brain, and neural progenitor cell (NPC) controls, including pairwise multiple-comparison analyses. * Adj. p-value <0.05, ** Adj p-value <0.001

Pairwise Comparisons of Subgroup Type

Sample 1 vs. Sample 2	Test Statistic	Std. Error	Std. Test Statistic	Sig.	Adj. Sig.[a]	
**Adult_brain-Fetal_brain**	-9.250	6.540	-1.414	.157	1.000	False
**Adult_brain-SHH**	28.042	7.619	3.680	<.001	.007	True
**Adult_brain-WNT**	29.375	10.341	2.841	.005	.126	False
**Adult_brain-GPR 3**	31.375	7.064	4.441	<.001	.000	True
**Adult_brain-GPR 4**	31.819	7.064	4.504	<.001	.000	True
**Adult_brain-NCPs cell culture**	-45.542	12.236	-3.722	<.001	.006	True
**Adult_brain-HFFs cell culture**	-47.042	16.674	-2.821	.005	.134	False
**Fetal_brain-SHH**	18.792	7.619	2.466	.014	.382	False
**Fetal_brain-WNT**	20.125	10.341	1.946	.052	1.000	False
**Fetal_brain-GPR 3**	22.125	7.064	3.132	.002	.049	True
**Fetal_brain-GPR 4**	22.569	7.064	3.195	.001	.039	True
**Fetal_brain-NCPs cell culture**	-36.292	12.236	-2.966	.003	.084	False
**Fetal_brain-HFFs cell culture**	-37.792	16.674	-2.266	.023	.656	False
**SHH-WNT**	1.333	11.055	.121	.904	1.000	False
**SHH-GPR 3**	-3.333	8.073	-.413	.680	1.000	False
**SHH-GPR 4**	-3.778	8.073	-.468	.640	1.000	False
**SHH-NCPs cell culture**	-17.500	12.845	-1.362	.173	1.000	False
**SHH-HFFs cell culture**	-19.000	17.126	-1.109	.267	1.000	False
**WNT-GPR 3**	-2.000	10.680	-.187	.851	1.000	False
**WNT-GPR 4**	-2.444	10.680	-.229	.819	1.000	False
**WNT-NCPs cell culture**	-16.167	14.624	-1.105	.269	1.000	False
**WNT-HFFs cell culture**	-17.667	18.499	-.955	.340	1.000	False
**GPR 3-GPR 4**	-.444	7.552	-.059	.953	1.000	False

**GPR 3-NCPs cell culture**	-14.167	12.524	-1.131	.258	1.000	False
**GPR 3-HFFs cell culture**	-15.667	16.887	-.928	.354	1.000	False
**GPR 4-NCPs cell culture**	-13.722	12.524	-1.096	.273	1.000	False
**GPR 4-HFFs cell culture**	-15.222	16.887	-.901	.367	1.000	False
**NCPs cell culture-HFFs cell culture**	1.500	19.621	.076	.939	1.000	False
Each row tests the null hypothesis that the Sample 1 and Sample 2 distributions are the same.<br>Asymptotic significances (2-sided tests) are displayed. The significance level is .050.						
a. Significance values have been adjusted by the Bonferroni correction for multiple tests.						

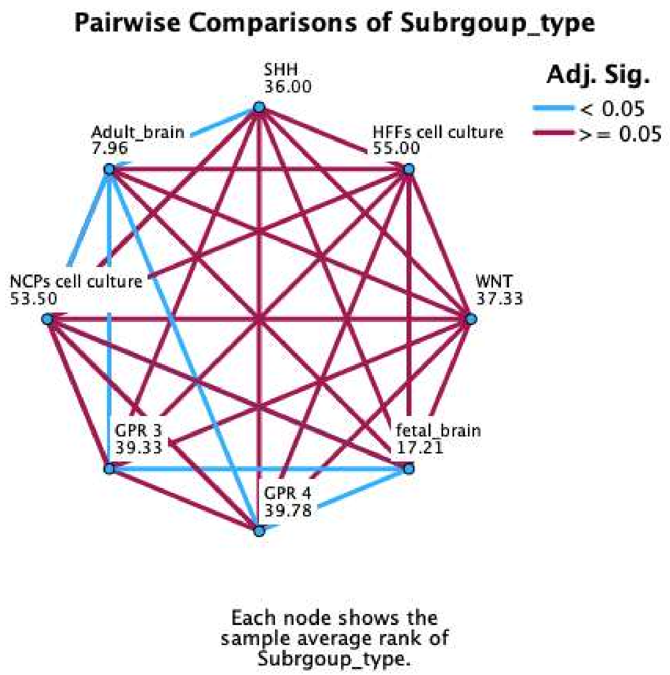

Supplement: Supplementary file 1 [file biomedicines-14-01328-s001.zip › Supplementary material S1.pdf]
