# Supplementary material for: Integrated Multi-Omics Analysis Reveals an HCMV-Associated Late-Gene Signature Associated with Poor Survival in Pediatric Group 3 Medulloblastoma
Source: Biomedicines. 2026 Jun 11;14(6):1328. doi: 10.3390/biomedicines14061328 (PMC13296392; doi:10.3390/biomedicines14061328)
Supplement: Supplementary file 1 [file biomedicines-14-01328-s001.zip › Supplementary material S2.pdf]

Supplementary material 2 - Differential expression of the HCMV late-gene signature across medulloblastoma molecular subgroups

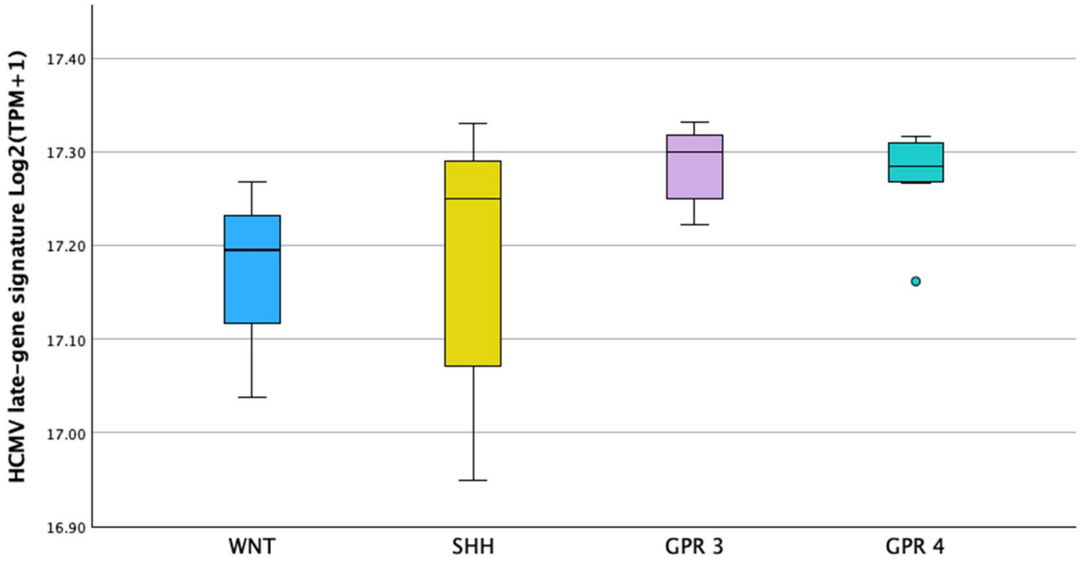

Kruskal–Wallis analysis of the composite HCMV late-gene signature (*UL76, UL88,* and *UL99*; $\log_2$(TPM + 1)) across WNT, SHH, Group 3 (GPR 3), and Group 4 tumors (GPR 4), with pairwise subgroup comparisons.

Pairwise Comparisons of Subgroup					
Subgroup vs. Subgroup	Test Statistic	Std. Error	Std. Test Statistic	*p*-value	Adj. *p*.[a]
WNT-SHH	−3.952	5.676	−0.696	0.486	1.000
WNT-GPR 4	−9.333	5.484	−1.702	0.089	0.533
WNT-GPR 3	−9.889	5.484	−1.803	0.071	0.428
SHH-GPR 4	−5.381	4.146	−1.298	0.194	1.000
SHH-GPR 3	−5.937	4.146	−1.432	0.152	0.913
GPR 4-GPR 3	0.556	3.878	0.143	0.886	1.000

Each row tests the null hypothesis that the Sample 1 and Sample 2 distributions are the same.
Asymptotic significances (2-sided tests) are displayed. The significance level is 0.050.

a. *p*-values have been adjusted by the Bonferroni correction for multiple tests.

Independent-Samples Kruskal-Wallis Test Summary	
Total N	28
Test Statistic	4.956[a]
Degree Of Freedom	3
Asymptotic *p-value*(2-sided test)	0.175

a. The test statistic is adjusted for ties.
