# Supplementary material for: Integrated Multi-Omics Analysis Reveals an HCMV-Associated Late-Gene Signature Associated with Poor Survival in Pediatric Group 3 Medulloblastoma
Source: Biomedicines. 2026 Jun 11;14(6):1328. doi: 10.3390/biomedicines14061328 (PMC13296392; doi:10.3390/biomedicines14061328)
Supplement: Supplementary file 1 [file biomedicines-14-01328-s001.zip › Supplementary material.pdf]

Supplementary material

Table S4. Survival outcomes according to medulloblastoma molecular subgroup in the combined cohort (n = 84).

MB Subgroups	PFS (mean)	PFS (median)	p-value	EFS (mean)	EFS (median)	p-value	OS (mean)	OS (median)	p-value
WNT	75.5	-	0.104	135.2	-	0.009*	-	-	
others	43.48	52		84.15	76		-	-	
SHH	48.53	76	0.767	90.77	76	0.773	106	-	0.957
Others	49.85	59		98.43	94		129.67	-	
GPR3	34.73	19	0.118	76.94	94	0.070	83.53	-	0.013*
Others	54.79	76		105.96	147		141.02	-	
GPR4	47.42	59	0.641	90.73	55	0.642	132.27	-	0.541
others	47.39	52		96.85	114		112.56	-	

Univariate survival analyses for progression-free survival (PFS), event-free survival (EFS), and overall survival (OS) were compared across WNT, SHH, Group 3 (GPR3), and Group 4 (GPR4) medulloblastomas, as well as all other tumors in the cohort. The log-rank test was used to determine statistical significance.
**p-values* below 0.05 were deemed statistically significant.
p-values were derived from the log-rank test.

Table S5. Survival outcomes of HCMV-high molecular subgroups in the combined cohort (n = 84).

MB Subgroups	PFS (mean)	PFS (median)	p-value	EFS (mean)	EFS (median)	p-value	OS (mean)	OS (median)	p-value
HCMV-high WNT	43.67	-	0.590	84.0	84	0.452	-	-	
others	49.19	59		95.97	114				
HCMV-high SHH	20.5	6	0.442	25.67	20	0.253	-	-	
Others	50.16	59		98.3	114				
HCMV-high GPR3	32.5	12	0.065	60.11	14	0.001*	64.89	-	<0.001*
Others	53.47	76		105	129		137.61	-	
HCMV-high GPR4	41.18	18	0.688	43.18	39	0.050	67.9	-	0.546
others	50.21	59		105.43	129		126.7	-	

Univariate survival analysis comparing progression-free survival (PFS), event-free survival (EFS), and overall survival (OS) across HCMV-high WNT, SHH, Group 3 (GPR3), and Group 4 (GPR4) tumors versus all other cases in the combined cohort.
**p-values* below 0.05 were deemed statistically significant.
p-values were derived from the log-rank test.

Figure S1. Survival outcomes of HCMV-high molecular medulloblastoma subgroups in the combined cohort (n = 84).

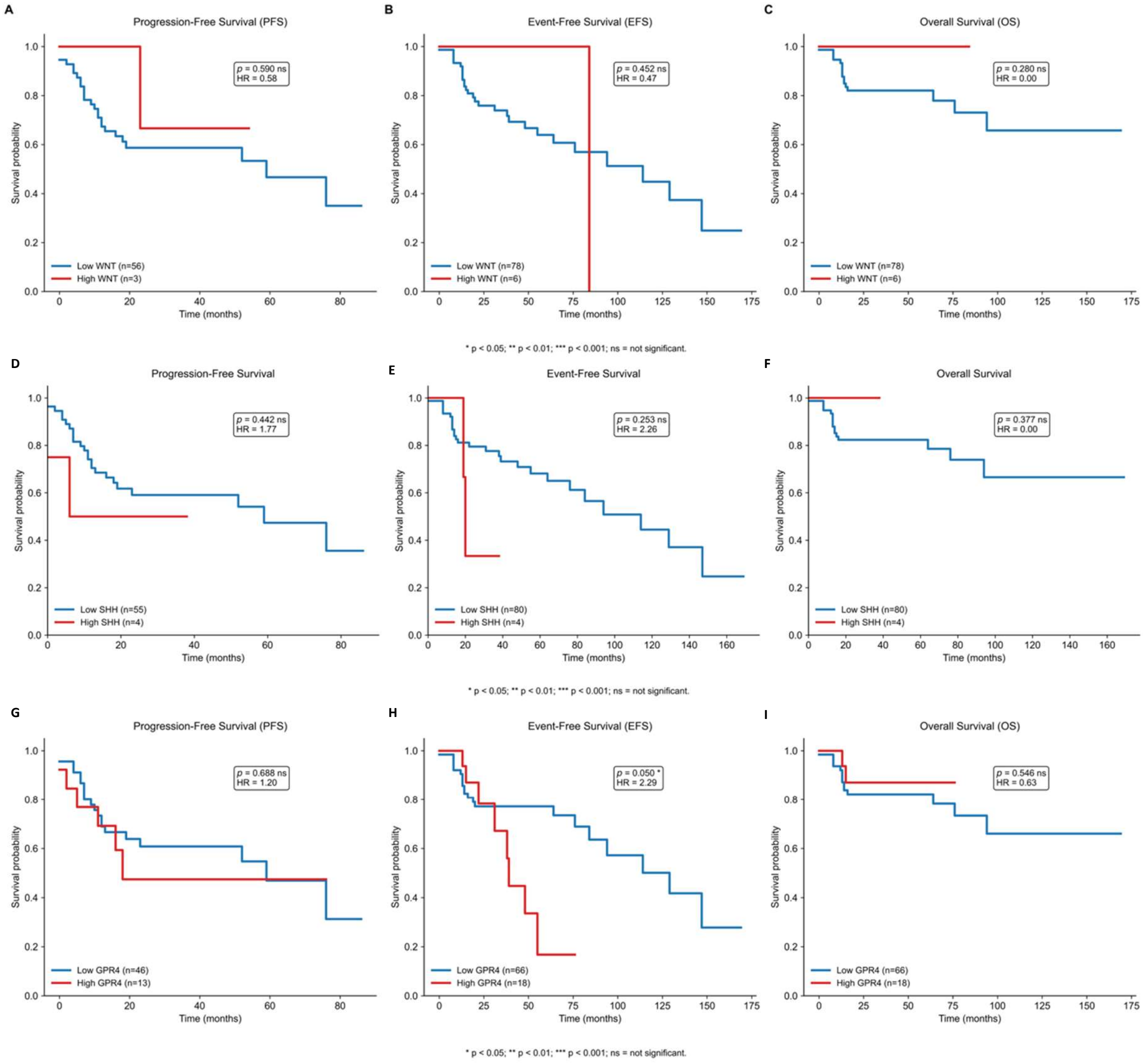

The upper, middle, and lower panels show survival comparisons for HCMV-high WNT (A–C), SHH (D–F), and Group 4 tumors (G–I), respectively, against all remaining tumors in the cohort. Corresponding survival statistics are presented in Supplementary Table S5.

Abbreviations: HCMV, human cytomegalovirus; TPM, transcripts per million; WGS, whole-genome sequencing; RNA-seq, RNA sequencing; PFS, progression-free survival; EFS, event-free survival; OS, overall survival; NPC, neural progenitor cell; SD, standard deviation.
